# Mechanisms of volatile organic compounds from bat cave environments against *Pseudogymnoascus destructans in vitro*

**DOI:** 10.1128/aem.01187-25

**Published:** 2025-11-13

**Authors:** Zihao Huang, Mingqi Shan, Aoqiang Li, Kangyu Wang, Zizhen Wei, Mingqi Shen, Jiaqi Lu, Keping Sun, Zhongle Li, Jiang Feng

**Affiliations:** 1 College of Life Science, Jilin Agricultural University 85112, Changchun, China; 2 School of Life Sciences, Central China Normal University 12446, Wuhan, China; 3 Jilin Provincial Key Laboratory of Animal Resource Conservation and Utilization, Northeast Normal University 47821, Changchun, China; 4 Jilin Provincial International Cooperation Key Laboratory for Biological Control of Agricultural Pests, Changchun, China; Universidad de los Andes, Bogotá, Colombia

**Keywords:** bats, pathogen, *Pseudogymnoascus destructans*, volatile organic compounds (VOCs), antifungal mechanisms, transcriptome, metabolome

## Abstract

White-nose syndrome, caused by the psychrophilic fungus *Pseudogymnoascus destructans,* is a wildlife disease that infects hibernating bats, resulting in the deaths of millions of bats in North America. Previous studies have confirmed that volatile organic compounds (VOCs) effectively inhibit the growth of *P. destructans*, but the antifungal mechanisms of these compounds have not been comprehensively characterized. This study screened two VOCs, 2,5-dimethylcyclohexanol (DMCH) and nonanal, identified from bat cave environments for their potent *in vitro* inhibition of *P. destructans*. Scanning and transmission electron microscopy revealed mycelial deformations and disruptions in cellular structures following treatment with these compounds. Physiological and biochemical assays showed higher Annexin V–fluorescein isothiocyanate/propidium (Annexin V–FITC/PI) signals consistent with mycelial apoptosis, increased reactive oxygen species (ROS) levels, higher adenosine triphosphate (ATP), superoxide anion, and glutathione (GSH) contents, and lower catalase (CAT) and superoxide dismutase (SOD) activities. Integrated transcriptomic and metabolomic analyses of mycelia exposed to DMCH or nonanal indicated disruption of cell wall and membrane integrity, altered expression of virulence-associated genes, and perturbation of primary metabolism and energy homeostasis. We also observed signatures of heightened oxidative stress, overexpression of ribosomal genes, and modulation of the MAPK signaling pathway. This study provides novel insights into the antifungal effects of VOCs targeting *P. destructans* and offers a scientific basis for combating white-nose syndrome.

**IMPORTANCE:**

White-nose syndrome, driven by the cold‐adapted fungus *Pseudogymnoascus destructans*, has decimated hibernating bat populations across North America, with profound ecological and economic consequences. Although volatile organic compounds (VOCs) have emerged as promising antifungal agents, their modes of action against *P. destructans* remain poorly defined. In this study, we demonstrate that two cave‐derived VOCs, 2,5-dimethylcyclohexanol (DMCH) and nonanal, not only deform fungal ultrastructure and trigger apoptosis, but also induce severe oxidative stress, disrupt energy metabolism, and dysregulate critical signaling pathways. By integrating transcriptomic and metabolomic profiling, we elucidate how DMCH and nonanal exposure compromises cell wall and membrane integrity, alters virulence gene expression, and perturbs the MAPK cascade, culminating in fungal cell death. These findings advance our mechanistic understanding of VOCs antifungal activity and highlight a novel, environmentally inspired strategy for mitigating white‐nose syndrome. Moreover, our work lays the groundwork for the development of VOC-based interventions to protect vulnerable bat populations and preserve ecosystem health.

## INTRODUCTION

Emerging infectious diseases have increased considerably in wildlife populations, imposing major burdens on the global economy and public health (1, 2). Notably, infectious diseases caused by fungal pathogens, including white-nose syndrome (WNS) in bats, saprolegniasis in fish, snake fungal disease, and chytridiomycosis in amphibians, have posed unprecedented threats to species extinctions (3–6). These diseases pose significant challenges to wildlife conservation due to their rapid onset, high mortality rates, and unknown pathogenesis.

White-nose syndrome, an emerging infectious disease affecting hibernating bats, is caused by the psychrophilic fungus *Pseudogymnoascus destructans* (7). Since 2006, WNS has killed millions of bats in North America, infecting multiple species (8, 9). However, bats have not been severely affected by WNS in all areas, showing notably lower fungal loads in China than in North America and no reported deaths of infected bats (10, 11). These differences may be due to host immune function, active substances metabolized by skin microorganisms that affect *P. destructans* growth, or environmental conditions limiting contact between *P. destructans* conidia and bats (12, 13). Indeed, the load and prevalence of *P. destructans* in bats have been shown to be related to environmental levels of the fungus, not the size of the bat colony (11). Therefore, it is necessary to investigate whether other microorganisms or compounds in the habitats of Chinese bat populations compete with *P. destructans* for resources or inhibit its growth.

Various biological, physical, and chemical agents, such as antifungals (14), microbial antagonists (15), vaccines (16), and electrolyte supplements (17), have been developed or tested for their effectiveness against *P. destructans* or in treating white-nose syndrome. However, due to the complexity of bat cave environments, the vulnerability of hosts, and the environmental impact of these strategies, volatile organic compounds (VOCs) are currently one of the most widely studied and intriguing control measures. For instance, low molecular weight VOCs, such as *trans*-2-hexenal, 2-methyl-1-butanol, and 1-pentanol, have been shown to exhibit rapid fungicidal effects (18, 19). Additionally, VOCs produced by *Rhodococcus rhodochrous* isolated from bat habitat soils effectively inhibited the growth of *P. destructans* (20). Gaseous VOCs produced by *Muscodor crispans* strain B-23 were shown to inhibit the growth of *P. destructans in vitro* and have been explored as a potential approach to mitigate the decline in wild bat populations associated with WNS (21). Currently, research on the antifungal mechanisms of VOCs mainly focuses on plant pathogens, with key mechanisms including damaging cell walls and cell membranes, inducing oxidative stress and apoptosis (22), disrupting metabolism, and disrupting morphology and structure (23). By comparison, evidence in animal-pathogenic fungi remains scarce. A few reports show that specific VOCs inhibit *P. destructans* growth *in vitro* and downregulate virulence-associated genes. For example, the plant volatile *trans*-2-hexenal reduces the expression of selected proteases and antioxidant genes in *P. destructans* (18). Clarifying these mechanisms is essential to realizing the biocontrol potential of VOCs and advancing their practical application.

This study aimed to reveal the molecular mechanisms of volatile organic compounds from bat cave environments against *P. destructans in vitro*. Soil and atmosphere samples were collected from bat caves in Northeast China, and VOCs were detected using gas chromatography–mass spectrometry. Additionally, the effects on *P. destructans* gene expression and metabolite contents were analyzed using transcriptomics and metabolomics. Meanwhile, the physiological and biochemical changes in *P. destructans* mycelia exposed to the compounds were examined. This study lays a critical theoretical foundation for the practical use of VOCs, providing a scientific basis for the feasibility of VOC-mediated *in situ* treatment of bats suffering from WNS.

## MATERIALS AND METHODS

### Sample collections from the field

**Fig 1:**
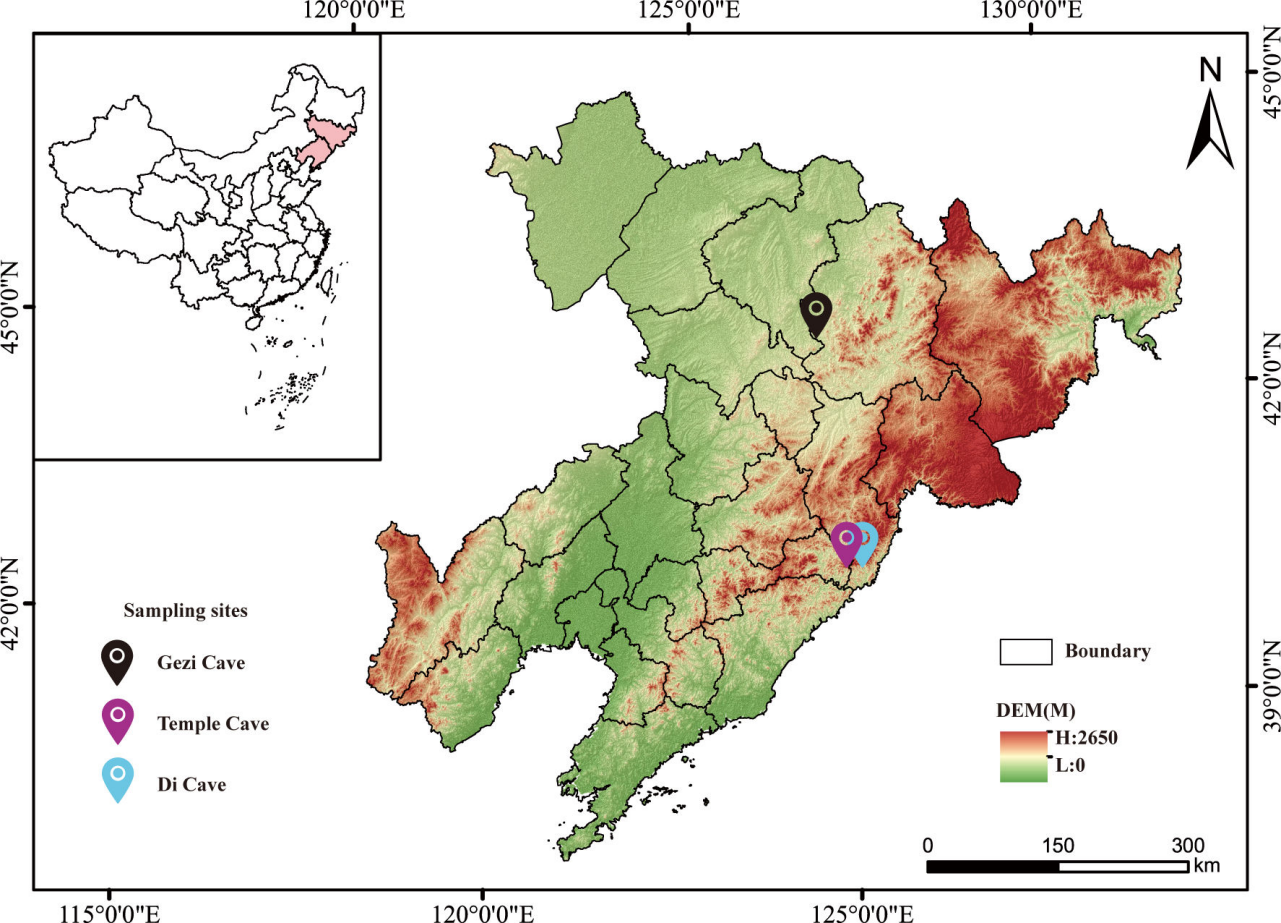
Map of the environmental sampling sites in bat caves in Northeast China. The map was created using ArcGIS. The underlying digital elevation model (DEM) data were obtained from the Geospatial Data Cloud website (https://www.gscloud.cn/), which is a publicly accessible platform providing open data resources.

Environmental soil (*n* = 16) and atmospheric (*n* = 22) samples (Table S1) were collected in early April 2021 from three caves in Northeast China with *P. destructans* loads where bats roost year-round: Temple Cave (Liaoning Province), Di Cave (Jilin Province), and Gezi Cave (Jilin Province) (12) (Fig. 1). Soil samples of approximately 5–10 g per sample were collected using a sterile shovel from the surface layer (5–10 cm) and stored in 100 mL sterile bottles. Cave atmospheric samples were collected using a vacuum gas sampler (BKMAM, China), with the sampler set to a rate of 3 L/min, and then stored in 3 L aluminum foil gas bags (BKMAN, China) at 13°C, protected from light, and analyzed by gas chromatography–mass spectrometry (GC–MS, Agilent, USA) within 24 h of collection. Bags were pre-flushed twice with cave air before sampling. All sampling devices were sterilized either under UV light exposure for 60 min or by autoclaving (121°C for 20 min) to prevent cross-contamination.

### Identification and screening of volatile organic compounds (VOCs)

Headspace VOCs were sampled by headspace solid-phase microextraction (HS-SPME) using a 65 µm PDMS/DVB StableFlex fiber (Supelco, USA). Samples were equilibrated at 40°C. The fiber was then exposed to the headspace for 40 min and thermally desorbed in the GC inlet at 250°C for 30 s. GC–MS was performed on an Agilent 5975 MSD operating in full-scan mode (m/z 50–550) with a DB-5-type capillary column (30 m × 0.25 mm × 0.25 µm, Agilent, USA). The inlet and transfer line were maintained at 250°C. Compounds were annotated by spectral matching to the NIST 2008 library (minimum match score ≥850), and relative abundances were calculated by peak-area normalization. Active compounds were homogenized using the Bray-Curtis distance matrix and visualized with non-metric multidimensional scaling (NMDS). Subsequently, differences in VOCs across samples were identified using non-parametric multivariate analysis of variance (PERMANOVA) with the *adonis* function in the R package *vegan* (24). Compounds with relative abundances greater than 1% were screened to identify both common and unique components among the samples. Subsequently, common components were tested for antifungal activity using agar plate challenge assays with *P. destructans*.

Three-week-old *P. destructans* was soaked in 10 mL of 1× phosphate-buffered saline with Tween 20 (PBST_20_). Conidial suspension was then collected by gentle scraping and filtered through sterile cotton to remove hyphal fragments. One hundred microliters of 2 × 10^5^ spores/mL *P. destructans* conidia (counted using a hemocytometer) was inoculated on sabouraud dextrose agar (SDA, Difco, USA), and then 100 µL of VOCs were inoculated on sterile antibiotic discs (6 mm, BKMAN, China) placed in the lids of the inverted plates. Plates were then sealed with parafilm and incubated at 13°C with 85% relative humidity for 14 days (12). Compounds that exhibited 100% inhibition of *P. destructans* hyphal growth and spore germination were selected to determine the minimum inhibitory concentration (MIC) and half-maximal inhibitory concentration (IC_50_), which were then used in subsequent experiments. The MIC and IC_50_ were determined by measuring the mycelial growth diameter on culture plates after 14 days of incubation. MIC is defined as the lowest VOC concentration that completely inhibits mycelial growth, where no visible growth of *P. destructans* is observed. IC_50_ is the VOC concentration that inhibits *P. destructans* mycelial growth by 50%. The inhibition rate (%) was calculated as inhibition rate % = (R1 − R2)/R1 × 100%, where R1 is the diameter of radial growth in the control group and R2 is the diameter of radial growth on the VOC-treated plate (25).

### Strain and chemicals

*Pseudogymnoascus destructans* strain JHCN111a (10) was provided by the Jilin Provincial Key Laboratory of Animal Resource Conservation and Utilization (Northeast Normal University, Changchun, China) and stored at −80°C. 2,5-Dimethylcyclohexanol (DMCH, CAS:3809-32-3, purity 99%) and nonanal (CAS:124-19-6, purity 96%) were procured from Macklin (Shanghai, China) and stored at 25°C. The superoxide dismutase (SOD) activity assay kit (AKAO001C), catalase (CAT) activity assay kit (AKAO003-1U), superoxide anion content assay kit (AKAO008C), ATP content assay kit (AKOP004U), reactive oxygen species (ROS) assay kit (AKCE002-1), and reduced glutathione (GSH) content assay kit (AKPR008C) were all purchased from Beijing Box Biotechnology Co., Ltd. (Beijing, China). The Annexin V–FITC/PI apoptosis detection kit (BB-4101) was obtained from Shanghai Beibo Biotechnology Co., Ltd. (Shanghai, China).

### Observations of morphology and ultrastructure of *P. destructans* mycelia

*P. destructans* mycelia treated with IC_50_ concentrations of DMCH and nonanal, along with untreated mycelia, were prepared for microtome sectioning according to the method of Sun et al. (26). In brief, mycelia were fixed overnight in 2.5% glutaraldehyde at 4°C and rinsed three times with PBS. After dehydration through a graded ethanol series, samples were vacuum freeze-dried. Dried specimens were sputter-coated with gold and imaged by scanning electron microscopy (SEM). For transmission electron microscopy (TEM), dehydrated samples were embedded in epoxy resin, microtomed into ultrathin sections, stained with uranyl acetate and lead citrate, and imaged. The micromorphology and ultrastructure of *P. destructans* mycelia were observed using a Zeiss Sigma 300 field emission SEM (Zeiss, Germany) and a TEM HT7800 (Hitachi, Japan), respectively.

### Assessment of apoptosis induction and reactive oxygen species (ROS) levels

*P. destructans* mycelia treated with IC_50_ concentrations of DMCH and nonanal, along with untreated mycelia, were collected and resuspended in 195 µL Annexin V–fluorescein isothiocyanate (Annexin V–FITC) binding buffer. Five microliters of Annexin V–FITC and 15 µL propidium iodide (PI) were then added, and the samples were incubated for 20 min at 4°C, according to the manufacturer’s instructions. During early apoptosis, phosphatidylserine (PS) in the cell membrane flips from the inner to the outer leaflet, and Annexin V binds specifically to PS, emitting green fluorescence. PI dye penetrates the cell membrane and binds to the nucleus, emitting red fluorescence during the middle and late stages of apoptosis. Apoptosis in the mycelia was observed under a confocal laser scanning microscope (CLSM, Leica, Germany). Similarly, to detect ROS levels, mycelia loaded with a 10 µM 2′,7′-dichlorodihydro-fluorescein diacetate (DCFH-DA) probe were incubated for 30 min at 37°C according to the kit instructions, and ROS accumulation in the mycelia was observed using CLSM. Non-fluorescent DCFH is oxidized by intracellular ROS to fluorescent DCF, and higher fluorescence indicates higher ROS levels.

### Biochemistry assays

Mycelia of *P. destructans* treated with IC_50_ concentrations of DMCH and nonanal, along with untreated mycelia, were collected and resuspended in the kit-supplied extraction buffer (with 10 mL of extract per g of mycelial mass), homogenized in an ice bath, and centrifuged at 10,000 × *g* for 20 min at 4°C. The supernatants were then placed on ice for further testing (27). Catalase (CAT) and superoxide dismutase (SOD) activities, as well as the contents of superoxide anions, adenosine triphosphate (ATP), and glutathione (GSH), were determined using respective kits according to manufacturers’ instructions.

### Transcriptome sequencing and analysis

Mycelia of *P. destructans* treated with IC_50_ concentrations of DMCH and nonanal, along with untreated mycelia, were collected, and total RNA was extracted using TRIzol (28). The concentrations, integrities, and DNA contamination of RNA samples were assessed as previously described (29). High-quality RNA was used to construct libraries using the Hieff NGS MaxUp Dual-mode mRNA Library Prep Kit for Illumina (YEASEN, Shanghai, China), and transcriptome libraries were then sequenced on the DNBSEQ-T7 platform. Sequence quality was evaluated using FastQC (30), and the raw data were processed with Trimmomatic to obtain clean reads (31). RNA-seq raw data are available in the NCBI SRA database under accession number PRJNA1097970.

Clean reads were aligned to the *P. destructans* reference genome sequence, and normalized gene expression levels were estimated in Transcripts Per Million (TPM) as calculated by String Tie (32). Subsequently, gene expression differences between groups were assessed using *DESeq2* (v1.12.4), with differentially expressed genes (DEGs) defined by |Fold Change| > 2 and False Discovery Rate (FDR) < 0.05 (33). Principal Component Analysis (PCA) was conducted to observe group clustering. Additionally, Kyoto Encyclopedia of Genes and Genomes (KEGG) functional analysis was performed using *clusterProfiler* (v3.0.5) (34). Significantly enriched KEGG pathways (*P* < 0.05) were then imported into OmicShare tools (OmicStudio, Guangzhou, China) for pathway-network visualization (35).

### Real-time quantitative polymerase chain reaction (RT-qPCR) analysis

RT-qPCR was performed to validate the RNA-seq results. Extracted total RNA was used to synthesize cDNA using the *TransScript* All-in-One First-Strand cDNA Synthesis SuperMix for qPCR (TransGen, Beijing, China), and RT-qPCR was performed using *PerfectStart* Green qPCR SuperMix (TransGen, Beijing, China) and qTOWER 3G (Analytic Jena, Jena, Germany). Primers were designed using Primer Premier 5.0 (Palo Alto, CA, USA), with EFG1 serving as the reference gene. Primer sequences for RT-qPCR are listed in Table S4. The relative expression levels of genes were calculated using the 2^-ΔΔCT^ method (36).

### Metabolome sequencing and analysis

Mycelia of *P. destructans* treated with IC_50_ concentrations of DMCH and nonanal, along with untreated mycelia, were collected for untargeted metabolomics analysis. Metabolites were extracted from the mycelia according to the method of Li et al. (2022) (37). Samples were analyzed using a Vanquish LC ultra-high performance liquid chromatograph (UHPLC) (Thermo Scientific, USA) coupled with Q Exactive HF mass spectrometry (MS) (Thermo Scientific, USA). Separation was performed on a HILIC column (2.1 mm × 100 mm, 1.7 µm, Waters, Ireland), with detection by electrospray ionization (ESI) in both positive and negative ion modes.

Raw MS data were converted to mzXML with ProteoWizard (38). Subsequently, XCMS was used for peak detection, retention-time correction, alignment, and peak integration (39). Metabolites were annotated by matching accurate mass (≤10 ppm) and, when available, MS/MS spectra to public libraries (Mass Bank, Metlin, HMDB, and MoNA). All annotations met MSI Level-2 or higher criteria. Multivariate analyses—principal component analysis (PCA), partial least squares discriminant analysis (PLS-DA), and orthogonal partial least squares discriminant analysis (OPLS-DA)—were performed in R using the *ropls* (v1.34.0) package (40). In addition, differentially expressed metabolites (DEMs) were identified based on the Student’s *t*-test (*P* < 0.05) and variable importance in projection (VIP >1) from the OPLS-DA model. Lastly, the KEGG database was used to explore the metabolic pathways of the differential metabolites (34, 41).

### Integrated analyses of transcriptome and metabolome data

The R package *MixOmics* (v6.26.0) (42) was utilized for multivariate dimensionality reduction analysis of the transcriptomic and metabolomic data. Correlation analysis between DEGs and DEMs was performed using the block splsda function and visualized with the plotVar and circosPlot functions. Relationships between genes and metabolites were also measured using regularized canonical correlation analysis (rCCA). DEGs and DEMs were then mapped to the KEGG database to identify pathways that co-vary at the transcriptional and metabolic levels.

### Statistical analyses

The biochemical and active compounds data were analyzed using SPSS 27.0 (IBM, USA). Shapiro-Wilk and Levene tests were employed for normal distribution and chi-square (χ²) tests, respectively. Tukey’s test was used to analyze significant differences in biochemical indices between treatment and control groups. Kruskal-Wallis and Wilcoxon rank sum tests were used to analyze differences in active compounds between groups, with *P* < 0.05 considered significant. Graphs were plotted using Origin 2018 (Origin Lab, USA). All experiments were performed in triplicate, and results were presented as mean ± standard deviation (SD).

## RESULTS

### Screening of VOCs in cave environments

A total of 272 VOCs were identified from 16 soil samples and 22 atmospheric samples using GC-MS. The NMDS cluster analysis, based on the Bray-Curtis dissimilarity matrix, revealed significant differences in VOCs between different sample types (NMDS with stress = 0.115, PERMANOVA, Pseudo-*F*_2, 38_ = 21.42, *P* = 0.001, *R*^2^ = 0.37), as well as between caves (Pseudo-*F*_3, 272_ = 1.98, *P* = 0.037, *R*^2^ = 0.1) (Fig. S1). In total, there were 40 compounds with relative abundances >1%, including 23 unique components in soil samples, 13 unique components in atmospheric samples, and 4 common components (Table S2). All common compounds were tested in agar plate challenge assays against *P. destructans*. Nonanal and DMCH exhibited the highest inhibition rates (100%) against *P. destructans* and were consistently present in various samples, which prompted their selection for further experiments. Their MIC values were 0.02 µL/mL and 0.55 µL/mL, respectively, while their IC_50_ values were 0.006 µL/mL and 0.36 µL/mL, respectively.

### Microscopic effects of active compounds on *P. destructans* mycelia

**Fig 2:**
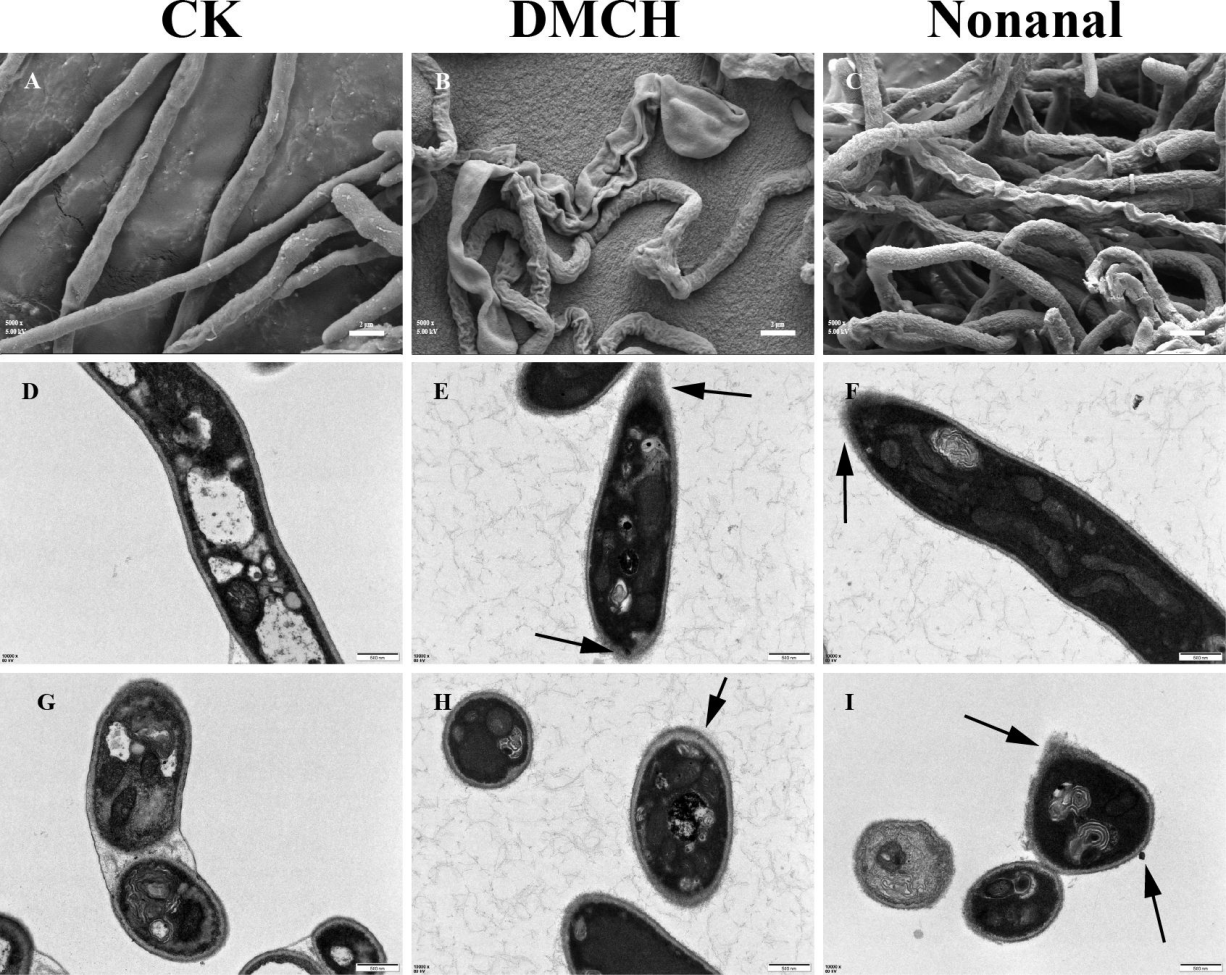
SEM and TEM images of *P. destructans* mycelia after treatment with compounds. (**A, D, G**) Untreated mycelia. (**B, E, H**) Mycelia treated with DMCH. (**C, F, I**) Mycelia treated with nonanal. Magnification 5,000× (A–C: bar 2 µm) and 10,000× (D–I: bar 500 nm). The arrows indicate damage to the hyphal cell wall and plasma membrane or leakage of cellular contents.

SEM analysis revealed that the mycelia of the control group were plump, smooth, and regular, with no damage to the cell walls or cell membranes (Fig. 2A). In contrast, the mycelia treated with both DMCH and nonanal exhibited signs of shrinkage, twisting, and contraction, particularly with partial mycelial breakage observed after nonanal treatment (Fig. 2B and C). TEM longitudinal sections of the mycelia in the control and treatment groups are shown in Fig. 2D through F, while transverse sections of the mycelia in the control and treatment groups are shown in Fig. 2G through I. The organelles of the control mycelia were intact and evenly distributed in the cytoplasm, with uniform thickness and normal cell wall and cell membrane morphologies. However, the mycelial cells in the treatment groups showed deformations, changes in cell wall thickness, disorganized cellular contents, and leakage.

### Effects of active compounds on apoptosis and intracellular ROS in *P. destructans* mycelia

**Fig 3:**
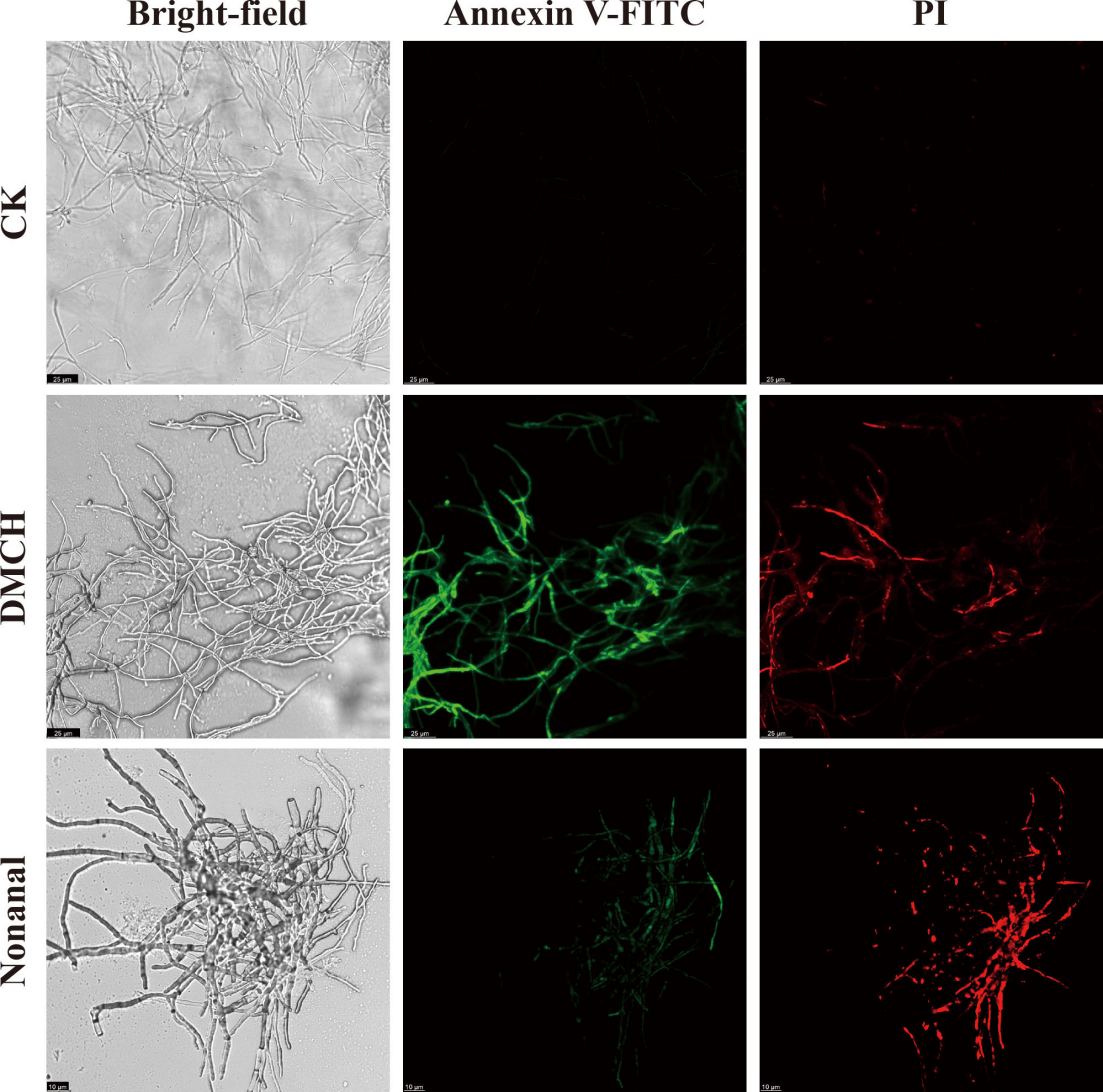
Fluorescence images of Annexin V–FITC/PI double-stained *P. destructans* mycelia after treatment with DMCH and nonanal observed with CLSM.

Annexin V–FITC/PI double staining and DCFH-DA assays were performed on *P. destructans* mycelia treated with active compounds using CLSM. Mycelia treated with DMCH and nonanal showed increased green (Annexin V–FITC) and red (PI) fluorescence relative to control (Fig. 3). ROS levels, assessed by DCFH-DA fluorescence intensity, were elevated in treated mycelia compared with control (Fig. S2).

### Effects of active compounds on biochemical properties of *P. destructans* mycelia

**Fig 4:**
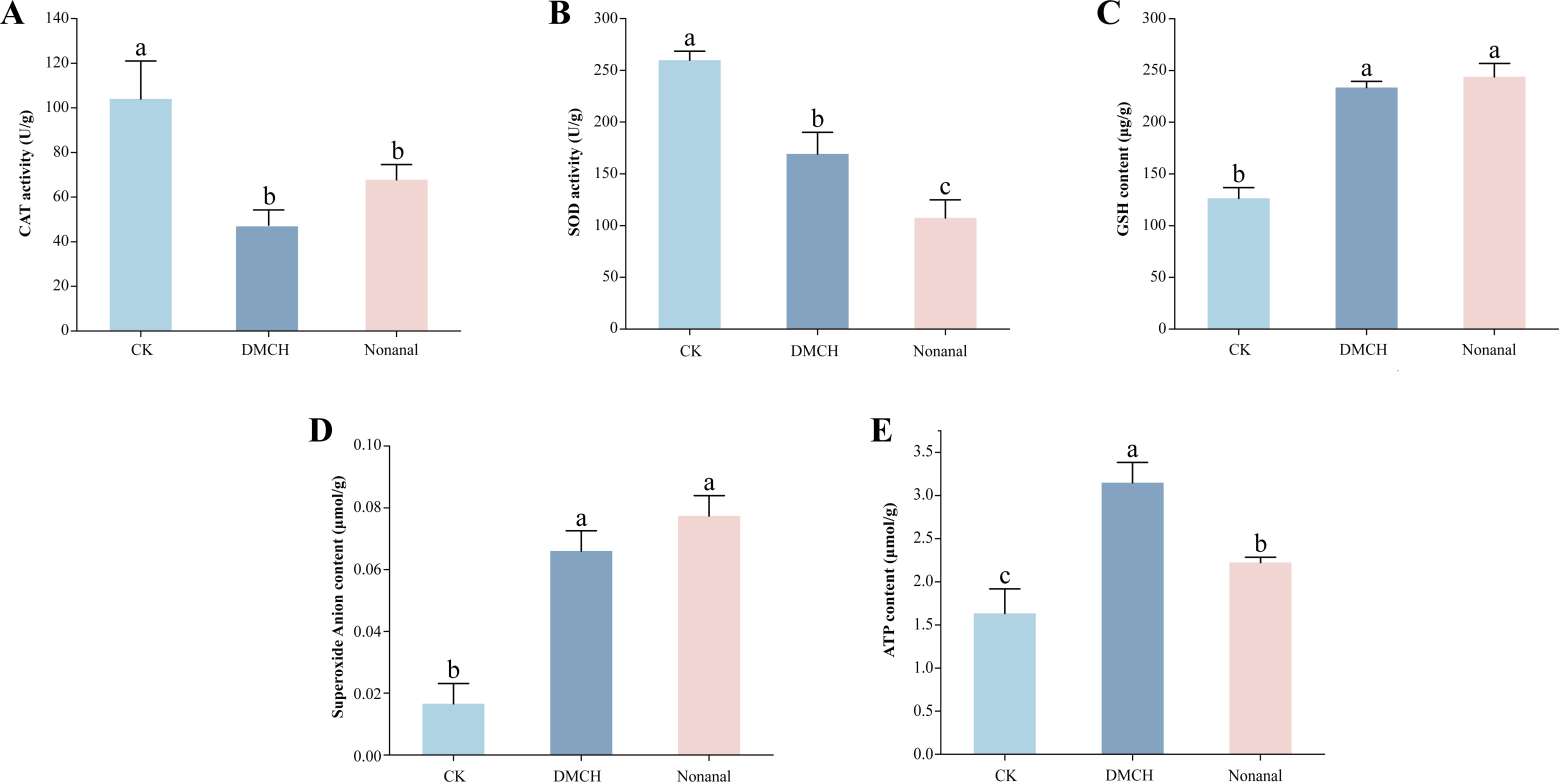
The changes in intracellular (**A**) CAT activity, (**B**) SOD activity, (**C**) GSH content, (**D**) superoxide anion content, and (**E**) ATP content in DMCH and nonanal treated *P. destructans* mycelia. Different letters indicate significant differences in biochemical parameters between groups (*P* < 0.05).

Mycelial superoxide anion contents were increased by 5.11- and 3.98-fold (Fig. 4D), and GSH contents were increased by 1.93- and 1.85-fold (Fig. 4C) in the nonanal and DMCH treatment groups, respectively, compared with the control group. Interestingly, CAT activity was reduced by 1.53- and 2.19-fold (Fig. 4A) and SOD activity by 2.42- and 1.54-fold (Fig. 4B) in nonanal and DMCH treatment groups, respectively. Intracellular ATP content increased by 1.93-fold (DMCH) and 1.36-fold (nonanal) relative to the control (Fig. 4E).

### Effects of active compounds on gene expression in *P. destructans* mycelia

**Fig 5:**
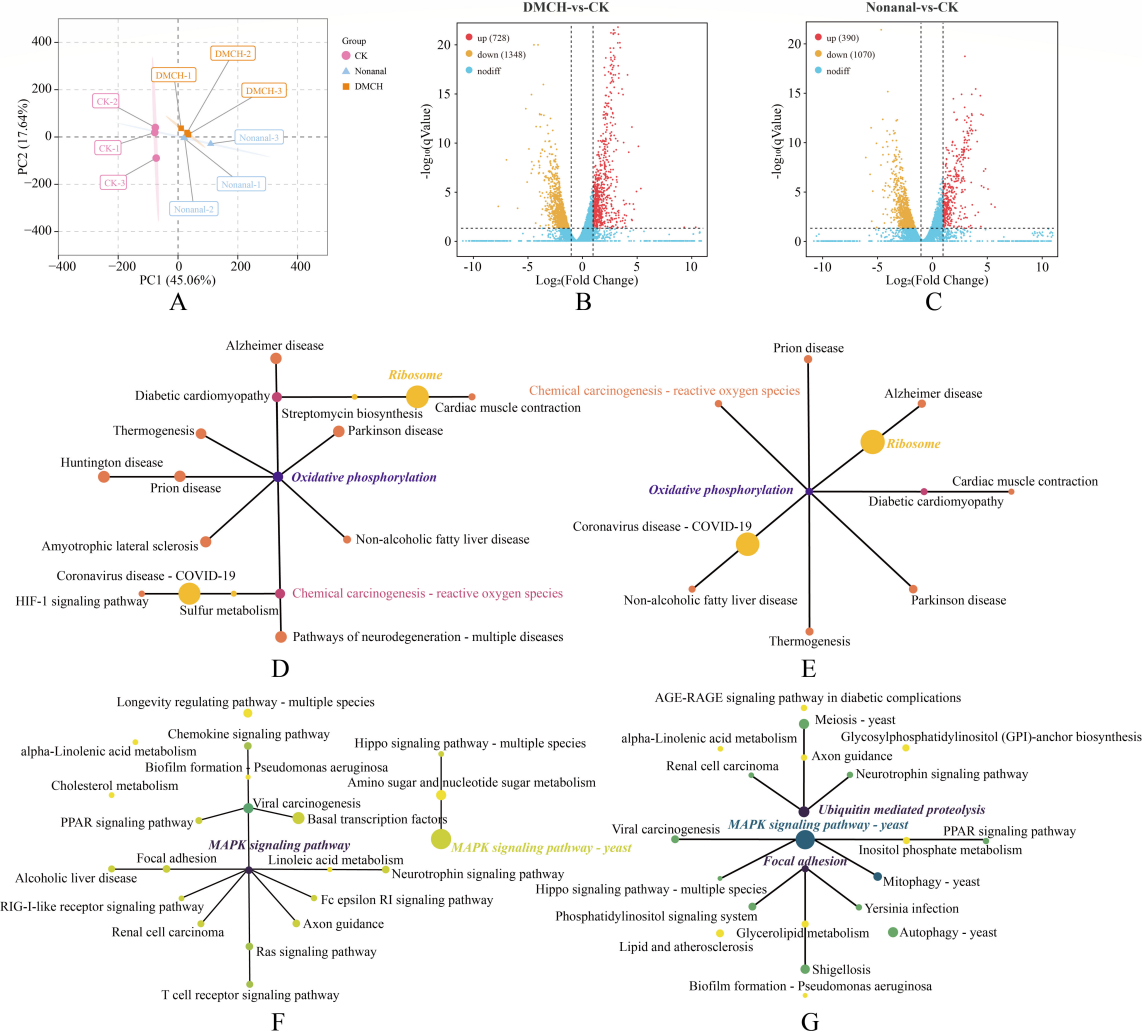
The transcriptomics analysis of *P. destructans* mycelia after treatment with DMCH and nonanal. (**A**) Principal component analysis of gene expression in *P. destructans* mycelia treated with DMCH and nonanal. (**B, C**) Volcano plots of upregulated, downregulated, and unchanged genes in the DMCH and nonanal groups, respectively. (**D, E**) Network analysis of KEGG-enriched pathways of upregulated DEGs in the DMCH and nonanal groups, respectively. (**F, G**) Network analysis of KEGG-enriched pathways of downregulated DEGs in the DMCH and nonanal groups, respectively. In the network diagrams, larger circles represent a greater number of genes enriched in the pathway, while darker colors indicate higher pathway connectivity.

To elucidate the molecular mechanisms of these active compounds against *P. destructans*, we analyzed the overall gene expression of both treated and untreated *P. destructans* mycelia using RNA-seq. Quality control statistics for RNA-seq reads are shown in Table S3, with Q30 and mapping rates higher than 95.24% and 93.59% for all samples. PCA showed clustering among control and treatment groups (Fig. 5A). To identify DEGs, cross-tabulation analyses were performed between treated and control groups, with 2,076 DEGs (728 upregulated and 1,348 downregulated) identified in the DMCH treatment and 1,460 DEGs (390 upregulated and 1,070 downregulated) identified in the nonanal treatment group (Fig. 5B and C). Potential *P. destructans* virulence genes and protease-encoding genes identified in previous studies (43) were screened further (Table S5). We observed decreased expression of virulence genes, such as *Subtilisin-like protease 1* and *Subtilisin-like protease 2*.

To further investigate the biological pathways affected by the active compounds in *P. destructans*, we performed KEGG pathway annotation of the DEGs in the treatment group (Table S6). The KEGG pathway network built from significantly enriched pathways (*P* < 0.05) highlighted oxidative phosphorylation (ko00190), ribosome (ko03010), and the MAPK signaling pathway (yeast; ko04011) as high-degree hub nodes (Fig. 5D through G). Overall, DEGs were enriched in pathways related to energy metabolism, signal transduction, and protein synthesis.

### Key genes verification using RT-qPCR

To validate the RNA-seq data, nine representative DEGs (*CHS7*, *CHS1*, *CDC42*, *STE12*, *COX12*, *QCR8*, *RPS7*, *RPS3*, *SP1*) were quantified by RT-qPCR. As shown in Fig. S3, the RT-qPCR expression trends of these genes in both treatment groups were consistent with the RNA-seq results.

### Effects of active compounds on metabolites in *P. destructans* mycelia

**Fig 6:**
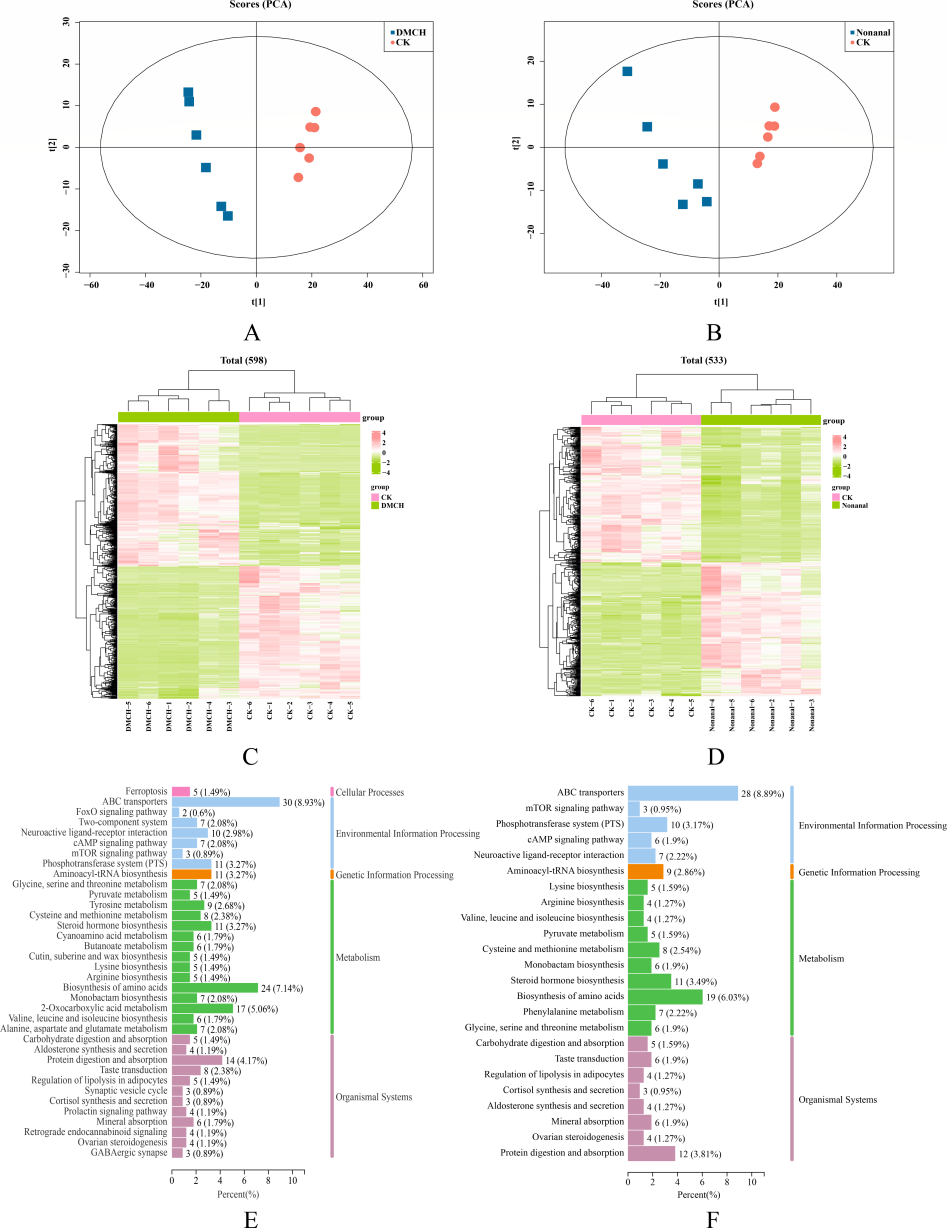
Metabolomics analysis of *P. destructans* mycelia after treatment with DMCH and nonanal. (**A, B**) Principal component analysis of metabolites in *P. destructans* mycelia treated with DMCH and nonanal, respectively. (**C, D**) Cluster analysis heat maps of DEMs of *P. destructans* mycelia treated with DMCH and nonanal, respectively. (**E, F**) KEGG enrichment analysis of DEMs of *P. destructans* mycelia treated with DMCH and nonanal, respectively.

To further investigate the impact of active compounds on *P. destructans* metabolites, metabolomic analysis was performed using UHPLC-MS/MS. PCA revealed significant differences between the control and both treatment groups (Fig. 6A and B). OPLS-DA analysis showed more distinct separation between the groups. Permutation tests validated the model, as the R2 and Q2 values of the random models gradually decreased, indicating no overfitting and demonstrating the model’s robustness (Fig. S4). Additionally, 2,069 metabolites were identified (1,161 in ESI^+^ mode and 908 in ESI^-^ mode). Among these, 598 DEMs were identified in the DMCH group and 533 in the nonanal group as compared with the control group (Fig. 6C and D).

KEGG enrichment analysis indicated that DEMs of both DMCH and nonanal-treated mycelia were involved in various metabolic pathways (Fig. 6E and F). Both DMCH and nonanal significantly interfered with pathways including ABC transporter, aminoacyl-tRNA biosynthesis, and amino acid biosynthesis. Specific details are provided in Table S7.

### Combined analysis of the transcriptome and metabolome

To further elucidate the molecular mechanisms of DMCH and nonanal against *P. destructans*, *MixOmics* was employed to explore the relationship between the transcriptome and metabolome. Concentric diagrams revealed that most DEGs and DEMs were far from the center of the circle, indicating a high correlation between them (Fig. S5A and B). Consistently, rCCA identified both positive and negative gene–metabolite associations (Fig. S4C and D).

Additionally, KEGG pathway enrichment analysis was conducted on DEGs and DEMs. The top 10 pathways with the highest number of genes and metabolites identified in the DMCH and nonanal groups, respectively, are illustrated in Fig. S6. Across both treatment groups, large numbers of differentially expressed molecules were involved in pathways including thermogenesis, oxidative phosphorylation, and amino sugar and nucleotide sugar metabolism.

## DISCUSSION

White-nose syndrome, caused by *P. destructans*, is considered one of the deadliest infectious diseases recorded in mammals (44). VOCs are key components of the antimicrobial activity of microbial agents, inhibiting the growth of pathogens in various ecosystems through contact-independent mechanisms and may further suppress their pathogenicity by reducing the expression of virulence factors (45). VOCs have been demonstrated to be effective in controlling *P. destructans in vitro* and may potentially reduce bats’ exposure to this pathogen in hibernation environments, as suggested by field observations (21). Therefore, our study aimed to identify the composition of VOCs and their interactions with *P. destructans in vitro* in bat caves across China. We integrated physiological and biochemical experiments along with transcriptomic and metabolomic assays to comprehensively characterize the molecular mechanisms of VOCs against *P. destructans.*

### Effects of DMCH and nonanal on cell wall and membrane synthesis in *P. destructans*

Electron microscopy revealed deformations, fractures, and cytoplasmic leakage in *P. destructans* mycelia after treatment with DMCH and nonanal, indicating that these compounds disrupted the cell wall and membrane structures of the mycelia, consistent with the mycelial deformation reported for *Fusarium graminearum* exposed to 2-phenylethanol (26). The fungal cell wall is a dynamic structure primarily composed of chitin, glucan, and mannan and is essential for cell viability, morphological maintenance, and pathogenesis (46). Chitin, a major structural component of fungal cell walls, is synthesized by chitin synthase (CHS), and disruption of CHS class I or III genes affected the cell walls and virulence of *Botrytis cinerea* (47). Transcriptome analysis revealed that the chitin synthase I gene *CHS1* (VC83_06323) and the chitin synthase III gene *CHS7* (VC83_05759) were downregulated after treatment with DMCH and nonanal. Similar CHS-associated (chitin synthase–associated) cell wall phenotypes have also been observed during bacterial antagonism (e.g., *Bacillus subtilis* Z-14 against *F. graminearum*) (48). Thus, we speculate that these active compounds may affect chitin synthesis and, consequently, the integrity of the cell wall.

Fungal cell membranes, which are targets for the antifungal activity of volatile plant components, are enriched with various lipids, including glycerophospholipids, sphingolipids, and sterols (49). Glycerophospholipids are major structural components of eukaryotic cell membranes, and their downregulation significantly disrupts membrane permeability and stability (50). In this study, glycerophospholipid metabolism-related metabolites, such as choline, PC (16:0/16:0), and glycerophospholipid choline, were downregulated in the treatment groups. Phosphatidylcholine (PC) is essential for maintaining cell membrane permeability, and its reduction has been shown to result in increased membrane permeability (51). Meanwhile, disruption of glycerophospholipid metabolism was accompanied by enhanced membrane lipid peroxidation. This pattern is consistent with membrane alterations reported for *Aspergillus flavus* after eugenol exposure and with reduced glycerophospholipid metabolism in *B. cinerea* upon exposure to 2-phenylethanol (25, 52). Additionally, the gene *ERG5* (VC83_01853), involved in ergosterol synthesis, was downregulated after DMCH treatment. The downregulation of *ERG5*, a target for antifungal drug development, resulted in the blockage of ergosterol synthesis. Perturbation of the ergosterol biosynthesis pathway has also been observed in *A. flavus* upon exposure to *trans*-anethole (53). These results indicated that DMCH and nonanal disrupted the integrity of *P. destructans* cell membranes.

### Effects of DMCH and nonanal on the virulence of *P. destructans*

For *P. destructans* and WNS, proteases have received the greatest attention as putative virulence factors (18). In this study, expression of many protease genes was significantly altered following treatment with active compounds (Table S4). This observation is consistent with prior studies showing that the plant volatile *trans*-2-hexenal downregulates virulence-associated genes in *P. destructans* (18). *Subtilisin-like protease 2* (*Pdsp1*) (VC83_06062) encodes the primary protease secreted by *P. destructans*. This collagen-degrading enzyme is thought to be associated with epidermal wing necrosis in WNS (54). *Subtilisin-like protease 1* (*Pdsp2*) (VC83_04892), which is highly similar to *Pdsp1*, was also downregulated in this study. Aspartyl family proteases are essential for *Candida glabrata* virulence, and ubiquitin-specific proteases are crucial for *F. graminearum* development and virulence (55). Additionally, metabolomic analyses indicated a decrease in riboflavin content in the mycelia of the treated groups. When infecting the host epidermis, *P. destructans* secretes substantial amounts of cytotoxic riboflavin, causing tissue necrosis (56). In summary, our findings are consistent with reports that VOCs modulate virulence-associated functions in *P. destructans*. However, they diverge from many studies of plant-pathogenic fungi, which emphasize membrane damage and oxidative stress as primary modes of action, rather than direct suppression of virulence factors (22). Taken together, these observations suggest that DMCH and nonanal inhibit fungal growth while modulating virulence-associated pathways.

### Effects of DMCH and nonanal on metabolic processes in *P. destructans*

Metabolic flexibility and adaptability are critical for successful pathogen colonization, infection, and initiation of disease symptoms in the host (57). Metabolomic analyses indicated that treatment with DMCH and nonanal affected several metabolic pathways in *P. destructans* mycelia, including ABC transporters and biosynthesis of amino acids. ATP-binding cassette (ABC) transporters are one of the largest and oldest protein superfamilies located in the cell membrane, utilizing energy generated from ATP hydrolysis to transport various substances across membranes (58). In the treatment groups, ABC-related metabolites, such as glutathione, lysine, and arginine, were upregulated, while xylitol, alginate, and riboflavin were downregulated in *P. destructans* mycelia. This suggested that the VOCs interfered with ABC transporter function, disrupting cell membrane integrity. These changes are consistent with reports that (*E*)−2-heptenal exposure triggers ABC transporter–mediated stress responses and disrupts membrane homeostasis in *A. flavus* (59). In the metabolomic analysis, multiple amino acids were found to be differentially expressed after treatment with VOCs, and the set of DEMs was significantly enriched for several amino acid biosynthesis pathways. Arginine is a precursor for nitric oxide biosynthesis and is essential for conidial germination in the filamentous fungus *Coniothyrium minitans* (60). Lysine biosynthesis also affects fungal growth (61). L-cysteine acts as an endogenous antioxidant in cells, upregulating defense genes by stimulating H_2_O_2_ production, thereby inhibiting grape powdery mildew (62).

Additionally, various metabolites related to sugar metabolism showed significant changes after treatment with VOCs, including a notable downregulation of trehalose and xylitol. Trehalose acts as a reserve carbohydrate, stress-protective molecule, and free radical scavenger, helping to prevent host cell damage (63). Furthermore, after DMCH treatment, genes related to the synthesis of trehalose-6-phosphate synthase (TPS1), such as VC83_06970, were downregulated. By contrast, (*E*)−2-heptenal exposure reportedly increases trehalose in *A. flavus* (59), suggesting species- or VOC-specific responses. These results suggested that DMCH and nonanal may affect the adaptability, pathogenicity, and growth of *P. destructans* by disrupting ABC transporters, interfering with amino acid biosynthesis, and altering sugar metabolism pathways.

### Effects of DMCH and nonanal on energy metabolism in *P. destructans*

**Fig 7:**
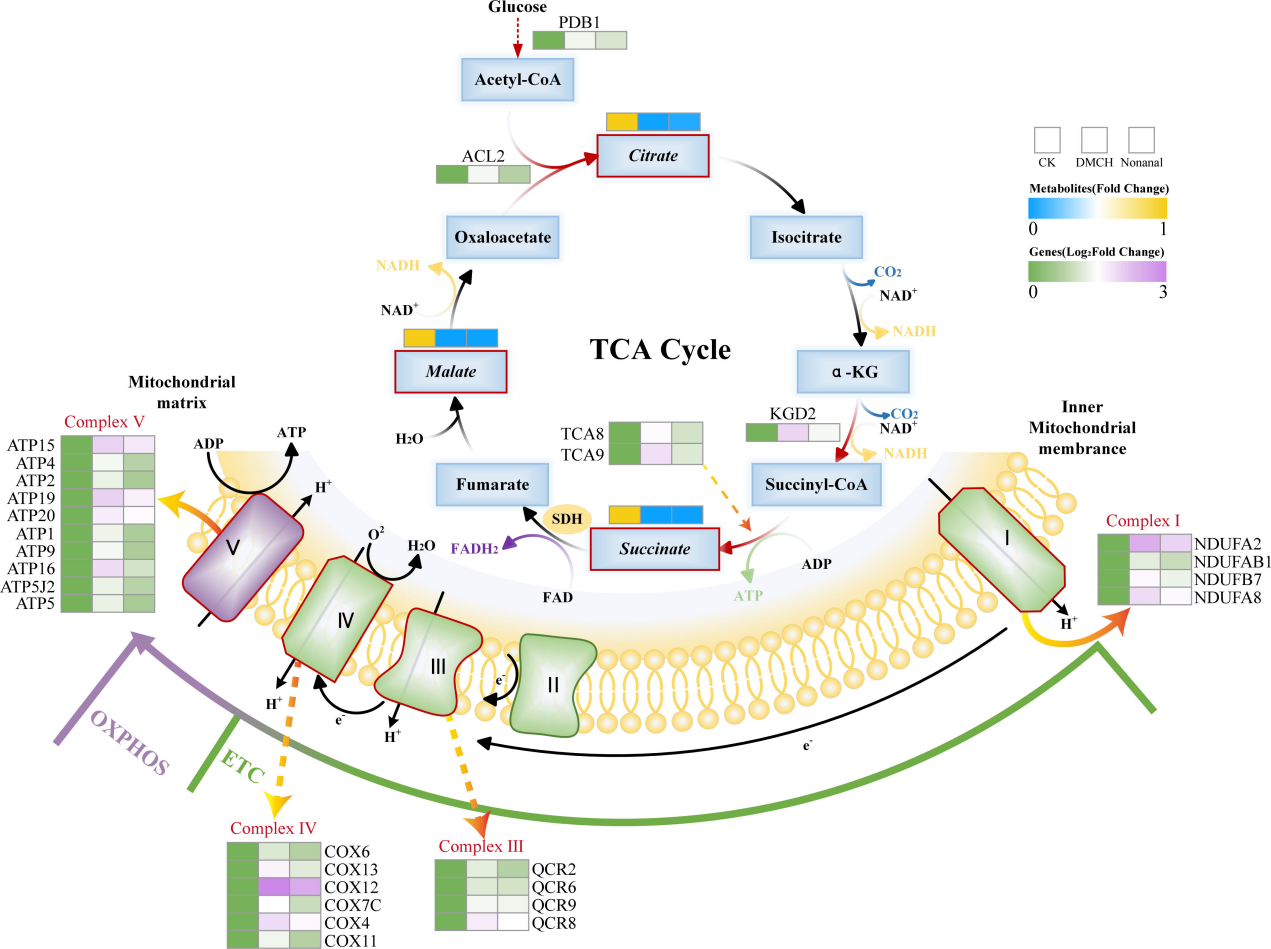
Energy metabolism disorders in *P. destructans* mycelia induced by DMCH and nonanal. Italics are used to distinguish between DEGs (*n* = 3) and DEMs (*n* = 6). Statistically significant changes in metabolites and gene expression are indicated by red boxes or arrows.

Most of the energy needed by eukaryotic cells is produced via the TCA cycle, which generates electron donors for oxidative phosphorylation (OXPHOS) to produce ATP (64). Succinate and malate are key intermediates in the TCA cycle, and their downregulation after VOC treatment may indicate damage to energy supply pathways. The succinyl-CoA gene (VC83_00878), whose protein product catalyzes the binding of succinate to CoA to produce ATP, was upregulated. The 2-oxoglutarate dehydrogenase complex gene (VC83_04627), involved in the oxidation of 2-oxoglutarate and providing energy to the TCA cycle, was also upregulated (Fig. 7). If the reducing equivalents from the TCA cycle are not fully utilized in OXPHOS, ROS production may increase, leading to oxidative damage (65, 66).

Transcriptomic analysis indicated that many of the ATP synthases (complex V) in the OXPHOS pathway were significantly upregulated (Fig. 7). Driven by protons, ATP synthase catalyzes the formation of ATP from adenosine diphosphate (ADP) and inorganic phosphate (Pi). Biochemical experiments showed an increase in ATP content in *P. destructans* mycelia treated with active compounds (Fig. 4E). Additionally, increased cytoplasmic ATP content is necessary for cell apoptosis (67). Annexin V–FITC/PI double staining showed enhanced red and green fluorescence in the mycelia of the treatment groups, indicating that the compounds induced cell apoptosis (Fig. 3). Lei et al. (53) found that the induction of apoptosis in *A. flavus* mycelia by *trans*-anethole was accompanied by an increase in ATP content (53). Therefore, we speculate that apoptosis in *P. destructans* mycelia induced by active compounds is mediated by high levels of OXPHOS and ATP synthesis.

### Effects of DMCH and nonanal on oxidative stress in *P. destructans*

Organisms continuously produce and eliminate ROS to maintain homeostasis, and disruptions to this balance result in oxidative stress (68). ROS production is highly regulated by the electron transport chain (ETC), where electron leakage during the reduction of oxygen to H_2_O by the ETC generates superoxide anions, which are precursors for ROS. Transcriptome analysis indicated that many genes in complexes I, III, and IV of the ETC were upregulated after treatment with VOCs (Fig. 7), indicating increased ETC activity. Biochemical experiments revealed an increase in superoxide anions in *P. destructans* mycelia following treatment with VOCs (Fig. 4D). Additionally, DCFH-DA staining showed enhanced green fluorescence and ROS accumulation in the mycelia of the treatment groups (Fig. S2), and the upregulated DEGs were significantly enriched for the chemical carcinogenesis-reactive oxygen species KEGG pathway, suggesting that the compounds induced ROS accumulation in *P. destructans* mycelia. This phenomenon is consistent with ROS accumulation in *B. cinerea* upon exposure to VOCs emitted by *Pseudomonas fluorescens* ZX (23). High levels of ROS have been shown to promote the release of apoptotic factors and initiate intrinsic apoptosis (69), which is consistent with the annexin V–FITC/PI double staining of VOC-treated mycelia (Fig. 3).

When ROS accumulate in excess, the body scavenges them using enzymes such as SOD and CAT, which comprise the earliest level of ROS-scavenging enzymes in cells (70). Treatment with VOCs resulted in downregulation of the expression of *CAT1* (VC83_01344) and *SOD1* (VC83_07077), consistent with biochemical experiments. Glutathione (GSH) also defends against intracellular oxidative damage. Metabolomic analyses and biochemical experiments indicated that GSH content increased in treated mycelia in response to oxidative stress. However, the extent of cellular oxidative stress depends on the balance between ROS production and scavenging. Although some antioxidant systems were activated, they could not completely counteract the effects of the compounds, similar to results reported by Zhang et al. (71). In summary, treatment with DMCH and nonanal enhanced ETC activity and superoxide anion levels and promoted ROS synthesis, leading to oxidative stress and impaired damage repair, thereby limiting mycelial growth.

### Effects of DMCH and nonanal on MAPK signaling in *P. destructans*

The MAPK signaling pathway is a highly conserved signaling system that enables fungi to sense and respond to environmental changes (72). In fungi, the MAPK pathway includes a three-component signaling relay consisting of MAPK kinase kinase (MAPKKK), MAPK kinase (MAPKK), and MAPK, which is involved in pheromone mating, cell wall integrity, response to high osmolarity, and filamentation growth (73). Previous studies have shown that the MAPK signaling pathway is crucial for the growth, development, reproduction, and pathogenicity of plant pathogens. Prior studies in *A. flavus* reported that exposure to certain volatiles (e.g., 1-octanol, perillaldehyde) is associated with reduced expression of MAPK signaling components (49, 74). In this study, many MAPK genes were downregulated after treatment with VOCs (Fig. S6), including *Bck1* (MAPKK) (VC83_09218) and *Ste7* (MAPKK) (VC83_008450). In fungi, three MAPK modules are activated by a specific agent, the P21-activated protein kinase (PKA) family member *Ste20* (VC83_01561), which was downregulated twofold after DMCH treatment. Ste20 is activated by the GTPase Cdc42, which further phosphorylates and activates Ste11 to function as a MAPKKK. *Cdc42* (VC83_05979) was downregulated in all treatment groups. These results suggested that DMCH and nonanal may block MAPK cascade initiation in *P. destructans* mycelia, disrupting its function and ultimately leading to cell cycle disruption and death.

### Effects of DMCH and nonanal on ribosomal protein expression in *P. destructans*

Ribosomes translate genetic information from mRNA into proteins, thereby regulating essential biological processes such as cell growth and differentiation (75). In eukaryotic cells, ribosomes are primarily composed of 40S small and 60S large subunits. Transcriptome analysis indicated that genes encoding small ribosomal subunit proteins, such as *PRS3* (VC83_05430) and *PRS1* (VC83_00544), and large subunit proteins, such as *RPL7* (VC83_05314) and *RPL5* (VC83_03181), were upregulated after treatment with VOCs. Meanwhile, KEGG analysis of the DEMs revealed significant changes in the aminoacyl-tRNA biosynthesis pathway in *P. destructans* mycelia. Aminoacyl-tRNA biosynthesis accurately matches amino acids with their corresponding anticodons on tRNAs, a critical step in protein synthesis (76). These translational signatures are consistent with reports that (*E*)-2-hexenal exposure disrupts protein-synthesis pathways in *B. cinerea* (77). Thus, DMCH and nonanal may interfere with protein synthesis in *P. destructans*, thereby affecting its growth.

Besides synthesizing proteins, ribosomes in eukaryotic cells are involved in activities such as apoptosis and the maintenance of genome integrity (78). Ribosomal protein S3 is dynamic, with the amino acid residues 16–25 being crucial for inducing apoptosis (79). Grapefruit extract has been reported to induce apoptosis in yeast cells by disrupting ribosomal protein L14-A (80). Elevated ATP content, ROS accumulation, upregulation of ribosome-associated proteins, and enhanced Annexin V–FITC/PI double-staining in VOC-treated *P. destructans* mycelia indicated that these compounds induced apoptosis.

Overall, DMCH and nonanal induced broadly similar physiological and biochemical, transcriptomic, and metabolomic responses in *P. destructans*. However, the compounds differed in effect size and pathway emphasis. At the biochemical level, SOD activity declined more with nonanal, whereas CAT activity was more strongly inhibited by DMCH (Fig. 4A and B). Under our conditions, the two compounds differentially affected antioxidant-related indices; the biological significance of these differences remains to be determined. At the omics level, the magnitudes of differential gene expression and metabolite changes were generally greater for DMCH than for nonanal (Fig. 5 and 6). Beyond the shared KEGG pathways, DMCH additionally engaged ancillary pathways, notably ergosterol biosynthesis (e.g., *ERG5*) and trehalose metabolism (e.g., *TPS1*). Taken together, DMCH and nonanal appear to share core mechanisms while exhibiting compound-specific differences in effect size and in the relative contributions of affected pathways.

As cave-derived VOCs that inhibit *P. destructans in vitro*, DMCH and nonanal merit consideration as potential candidates for environmental management of WNS. To support field translation, future work should: (i) quantify concentrations of DMCH and nonanal in cave air and substrates and relate these concentrations to environmental fungal burden; (ii) evaluate delivery approaches that maintain effective yet environmentally compatible concentrations in hibernacula (e.g., slow-release matrices or aerosolization) under realistic ventilation regimes; and (iii) systematically assess environmental persistence, degradation pathways, and non-target effects on cave microbiota and invertebrates, as well as potential effects on bats. These efforts will contribute to a deeper understanding of the ecological impact of VOCs and inform the development of effective management strategies for WNS.

### Conclusion

**Fig 8:**
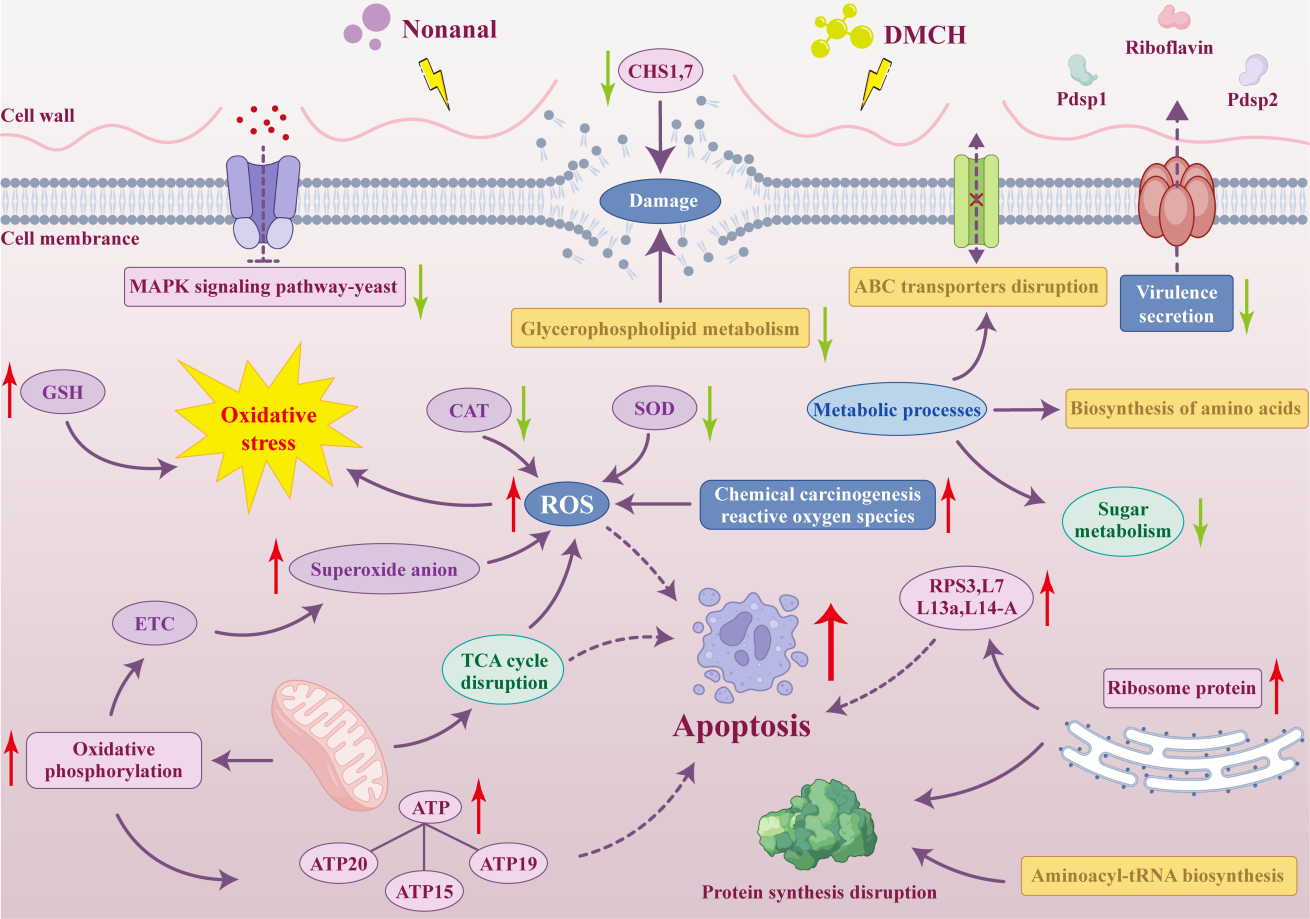
Model of molecular mechanisms of DMCH and nonanal inhibition of *P. destructans* growth.

In this study, we systematically investigated the mechanisms by which two VOCs, DMCH and nonanal, inhibit *P. destructans* growth using physiological and biochemical experiments, transcriptomics, and metabolomics. We demonstrated that DMCH and nonanal in bat cave environments effectively inhibited *P. destructans* growth *in vitro*. The inhibition of *P. destructans* mycelial growth upon exposure to DMCH and nonanal was accompanied by disrupting cell wall and membrane structures, impacting the expression of virulence genes, interfering with metabolic processes, disrupting the TCA cycle, causing high OXPHOS levels, inducing oxidative stress, overexpressing ribosomal proteins, interfering with the MAPK signaling pathway, and inducing apoptosis (Fig. 8). This study confirmed the fungicidal mechanism of VOCs and aimed to provide a scientific basis and potential applications for preventing and controlling white-nose syndrome, thus supporting the maintenance of healthy animal homeostasis.

## Data Availability

The data that support the findings of this study are openly available in Science Data Bank at https://doi.org/10.57760/sciencedb.14603. Transcriptome sequencing data were deposited into the NCBI Sequence Read Archive (SRA) under accession number PRJNA1097970. Metabolome raw data are openly available in Figshare at https://doi.org/10.6084/m9.figshare.27203106.v1.

## SUPPLEMENTAL MATERIAL

Supplemental material aem.01187-25-s0001.docx Fig. S1 to S7; Tables S1 to S7.

ASM does not own the copyrights to Supplemental Material that may be linked to, or accessed through, an article. The authors have granted ASM a non-exclusive, world-wide license to publish the Supplemental Material files. Please contact the corresponding author directly for reuse.
