# Supplementary material for: Mechanisms of volatile organic compounds from bat cave environments against Pseudogymnoascus destructans in vitro
Source: Appl Environ Microbiol. 2025 Nov 13;91(12):e01187-25. doi: 10.1128/aem.01187-25 (PMC12724331; doi:10.1128/aem.01187-25)
Supplement: Supplemental material — Fig. S1 to S7; Tables S1 to S7. [file aem.01187-25-s0001.docx]

**Mechanisms of volatile organic compounds** **from bat cave environments against *Pseudogymnoascus destructans* *in vitro***

Zihao Huang ^1^, Mingqi Shan ^1^, Aoqiang Li ^2^, Kangyu Wang ^1^, Zizhen Wei ^1^, Mingqi Shen ^1^, Jiaqi Lu ^1^, Keping Sun ^3^, Zhongle Li ^1,4,*^, Jiang Feng ^1,4,*^

^1^ College of Life Science, Jilin Agricultural University, Changchun 130118, China.

^2^ School of Life Sciences, Central China Normal University, Wuhan 430079, China.

^3^ Jilin Provincial Key Laboratory of Animal Resource Conservation and Utilization, Northeast Normal University, Changchun 130117, China.

^4^ Jilin Provincial International Cooperation Key Laboratory for Biological Control of Agricultural Pests, Changchun 130118, China.

* Corresponding authors: Zhongle Li, lzy1514316@126.com; Jiang Feng, fengj@nenu.edu.cn

**
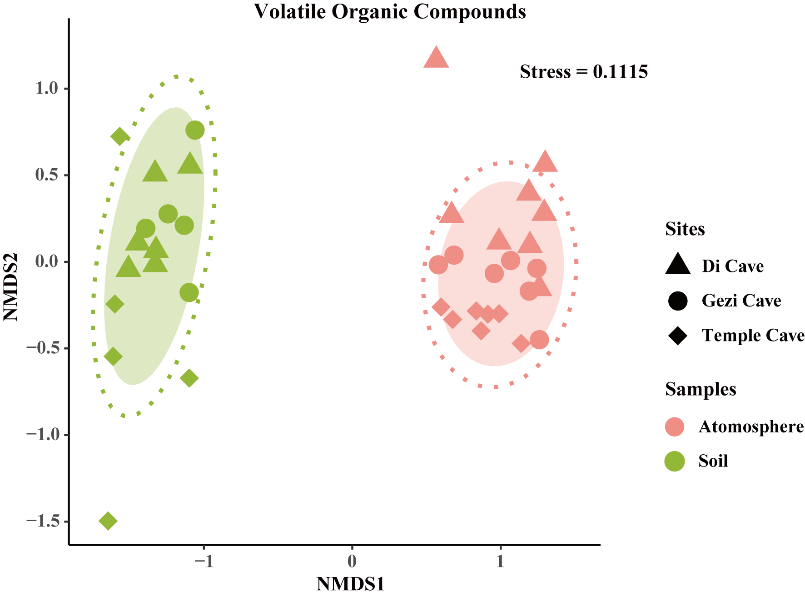
**

**Fig. S1.** Differences in volatile organic compounds among different caves and sample types. Non-metric multidimensional scaling analysis was based on Bray-Curtis distances.

**
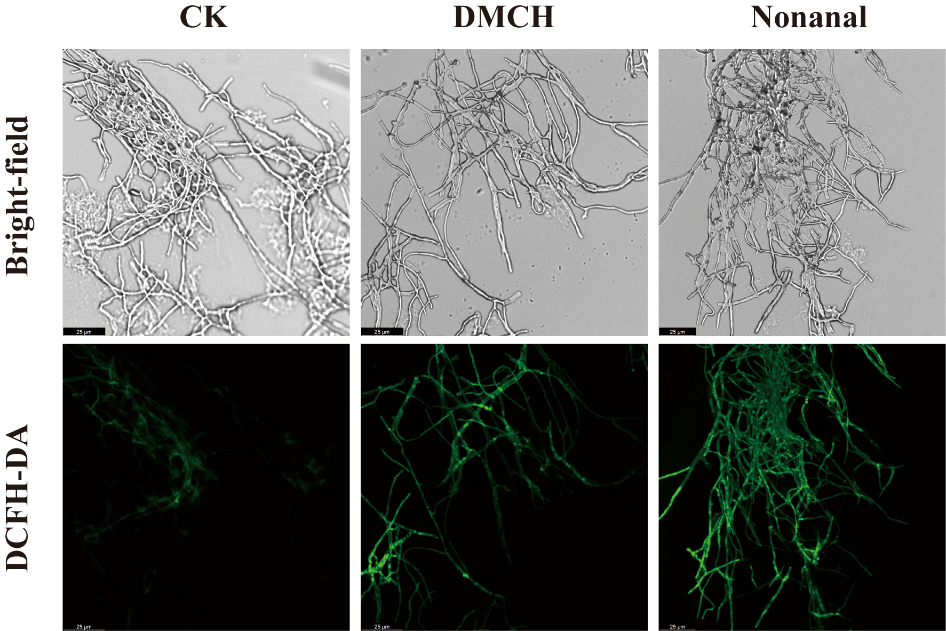
**

**Fig. S2.** Fluorescence images of DCFH-DA staining of *P.* *destructans* mycelia after treatment with DMCH and nonanal observed with CLSM.

**
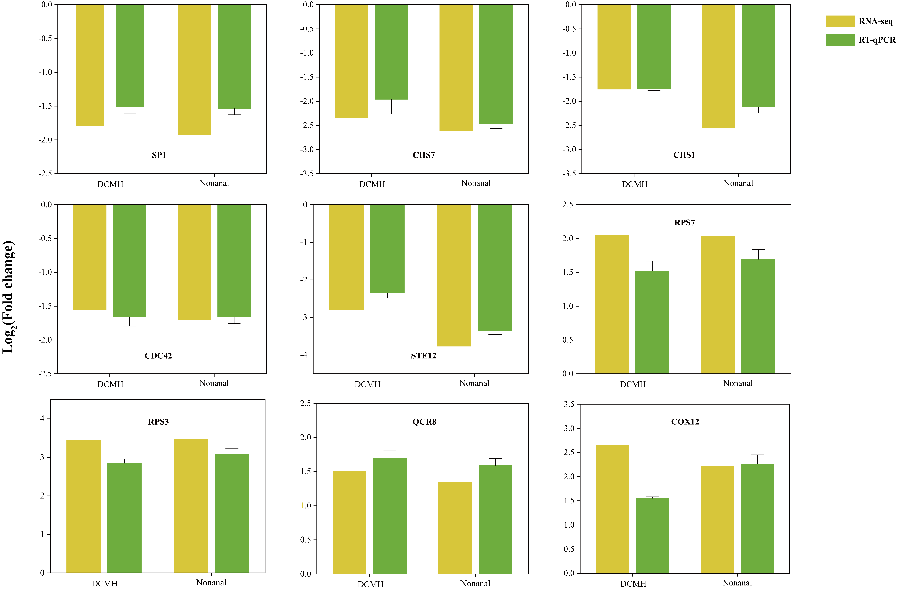
**

**Fig.** **S3.** Comparison of RNA-seq results (yellow) of representative DEGs with RT-qPCR results (green).

**
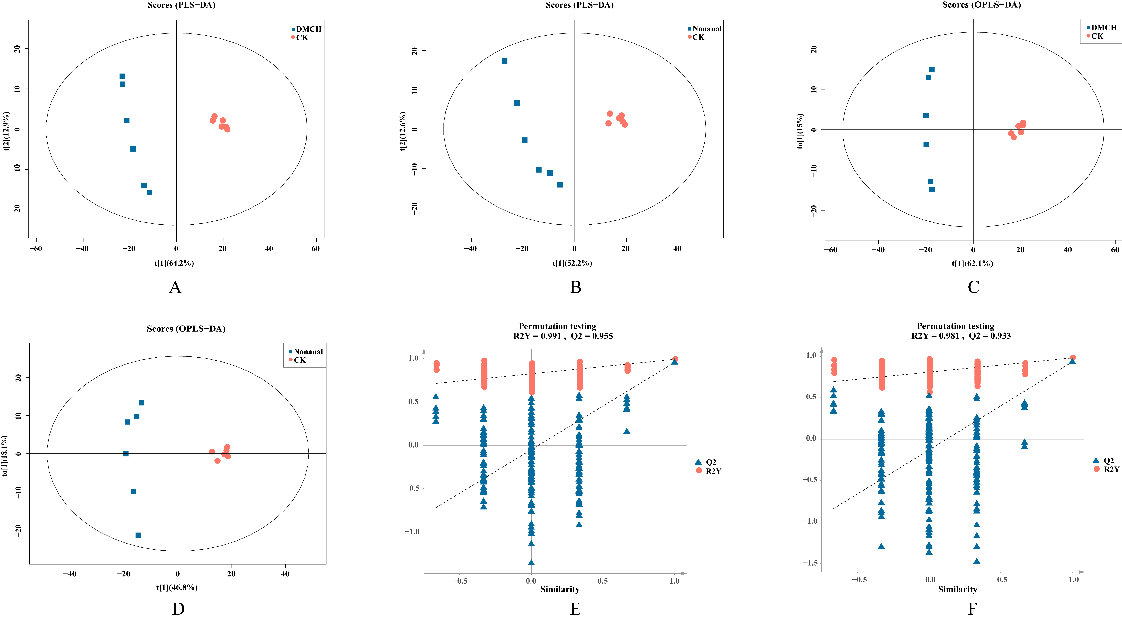
**

**Fig.** **S4.** Results of multivariate analysis of metabolome samples from DMCH and nonanal. (A, B) PLS-DA score plots for the DMCH and nonanal groups, respectively. (C, D) OPLS-DA score plots for the DMCH and nonanal groups, respectively. (E, F) Permutation plots of OPLS-DA for the DMCH and nonanal groups, respectively.

**
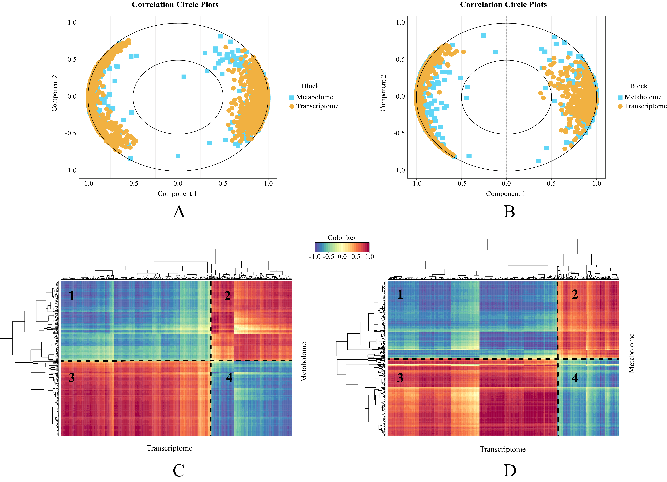
**

**Fig.** **S5.** Integrated analysis of transcriptomic and metabolomic data. (A, B) Concentric diagrams of the correlation of DEGs and DEMs between the CK group and the DMCH and nonanal groups, respectively. (C, D) Heatmap clusters of DEGs and DEMs for the DMCH and nonanal groups, respectively. Quadrants 1 and 4 represent DEGs with expression trends opposite to DEMs, indicating a negative correlation. Quadrants 2 and 3 represent DEGs with similar expression trends to DEMs, indicating positive regulation by metabolites.

**
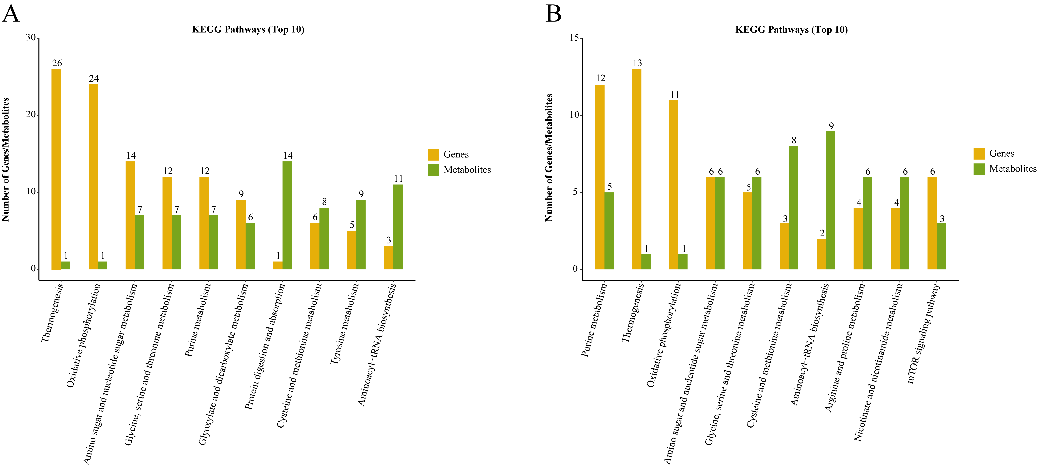
**

**Fig.** **S6.** Integrated KEGG pathways analysis of DEGs and DEMs. Top 10 KEGG pathways with the highest number of genes and metabolites identified for the (A) DMCH and (B) nonanal treatment groups.


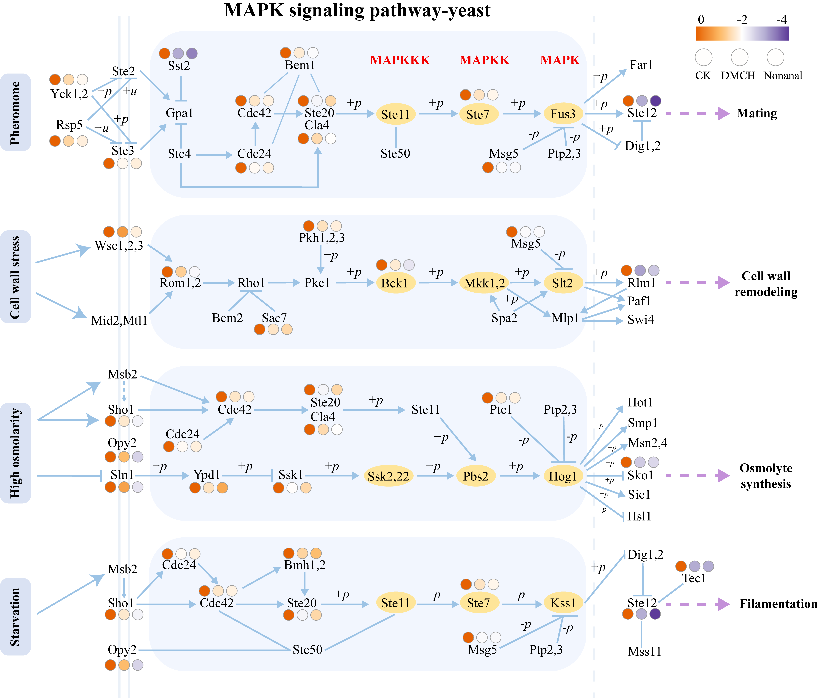


**Fig.** **S7.** Expression levels of Yeast-MAPK signaling pathway-related genes in *P. destructans* mycelia treated with DMCH and nonanal. Arrows and T-bars represent activation and inhibition, respectively, while dashed lines indicate indirect effects. The color scale represents gene expression levels based on TPM values.

**Table S1.** Field samples collection information.

| **Locality (province)** | **Types** | **Quantities** | **Distance (from entrance)** | **Bats abundance** | **Sampling date** |
| --- | --- | --- | --- | --- | --- |
| Di cave(Jilin) | Soil | 6 | Near (0-10m) | High (>20/m^2^) | 4/5/2021 |
| Di cave(Jilin) | Atmosphere | 8 | Near (0-10m) | High (>20/m^2^) | 4/5/2021 |
| Gezi cave(Jilin) | Soil | 5 | Far (30-50m) | Medium (10-20/m^2^) | 4/7/2021 |
| Gezi cave(Jilin) | Atmosphere | 7 | Far (30-50m) | Medium (10-20/m^2^) | 4/7/2021 |
| Temple cave(Liaoning) | Soil | 5 | Medium (10-30m) | Low (<10/m^2^) | 4/6/2021 |
| Temple cave(Liaoning) | Atmosphere | 7 | Medium (10-30m) | Low (<10/m^2^) | 4/6/2021 |

**Table S2.** Volatile organic compounds with relative abundance greater than 1%.

| **Compounds** | **CAS** | **Sources** | **Relative abundances** | **Features** |
| --- | --- | --- | --- | --- |
| Ethyl methyl carbonate | 623-53-0 | Soil/Atmosphere | 1.10%/16.74% | Common |
| Isovaleric acid | 503-74-2 | Soil/Atmosphere | 3.11%/6.88% | Common |
| Nonanal | 124-19-6 | Soil/Atmosphere | 3.36%/3.25% | Common |
| 2,5-Dimethylcyclohexanol | 3809-32-3 | Soil/Atmosphere | 2.53%/1.53% | Common |
| Cycloheptatriene | 544-25-2 | Soil/Atmosphere | 1.33%/1.00% | Soil-specific |
| Benzaldehyde | 100-52-7 | Soil/Atmosphere | 3.52%/0.77% | Soil-specific |
| Toluene | 108-88-3 | Soil/Atmosphere | 1.15%/0.00% | Soil-specific |
| Octanal | 124-13-0 | Soil/Atmosphere | 1.22%/0.10% | Soil-specific |
| Hexadecane | 544-76-3 | Soil/Atmosphere | 2.11%/0.08% | Soil-specific |
| 4-Methyl-2-pentanone | 108-10-1 | Soil/Atmosphere | 1.29%/0.06% | Soil-specific |
| 1,2-Xylene | 95-47-6 | Soil/Atmosphere | 1.07%/0.00% | Soil-specific |
| 2-Butanone | 78-93-3 | Soil/Atmosphere | 6.82%/0.00% | Soil-specific |
| Isovaleraldehyde | 590-86-3 | Soil/Atmosphere | 4.90%/0.00% | Soil-specific |
| 2-Methylbutyraldehyde | 96-17-3 | Soil/Atmosphere | 3.34%/0.00% | Soil-specific |
| Nitric acid heptyl ester | 20633-12-9 | Soil/Atmosphere | 2.96%/0.00% | Soil-specific |
| Triacetin | 102-76-1 | Soil/Atmosphere | 2.74%/0.00% | Soil-specific |
| 6-Methyl-6-hepten-2-one | 10408-15-8 | Soil/Atmosphere | 2.67%/0.00% | Soil-specific |
| 3-Methyl-2-pentanone | 565-61-7 | Soil/Atmosphere | 2.41%/0.00% | Soil-specific |
| 1-Nitroheptane | 693-39-0 | Soil/Atmosphere | 2.26%/0.00% | Soil-specific |
| 3-Methyl-1-butanol | 123-51-3 | Soil/Atmosphere | 1.94%/0.00% | Soil-specific |
| Nerylacetone | 3879-26-3 | Soil/Atmosphere | 1.59%/0.00% | Soil-specific |
| Nitric acid hexyl ester | 20633-11-8 | Soil/Atmosphere | 1.46%/0.00% | Soil-specific |
| 1-Nitrohexane | 646-14-0 | Soil/Atmosphere | 1.43%/0.00% | Soil-specific |
| 1-Nitropentane | 628-05-7 | Soil/Atmosphere | 1.33%/0.00% | Soil-specific |
| 1-Nitrobutane | 627-05-4 | Soil/Atmosphere | 1.20%/0.00% | Soil-specific |
| (S)-(-)-2-Methyl-1-butanol | 1565-80-6 | Soil/Atmosphere | 1.07%/0.00% | Soil-specific |
| 2-Methyl-1-propanol | 78-83-1 | Soil/Atmosphere | 1.01%/0.00% | Soil-specific |
| Diethyl carbonate | 105-58-8 | Soil/Atmosphere | 0.98%/19.44% | Atmosphere-specific |
| 2,4-Di-tert-butylphenol | 96-76-4 | Soil/Atmosphere | 0.00%/5.55% | Atmosphere-specific |
| Cyclopropane | 75-19-4 | Soil/Atmosphere | 0.00%/5.39% | Atmosphere-specific |
| Myristic acid | 544-63-8 | Soil/Atmosphere | 0.19%/3.71% | Atmosphere-specific |
| 3-Methyl-2-heptanone | 2371-19-9 | Soil/Atmosphere | 0.00%/3.28% | Atmosphere-specific |
| Methylcyclobutane | 598-61-8 | Soil/Atmosphere | 0.00%/3.09% | Atmosphere-specific |
| 2,2,4,6,6-pentamethyl-Heptane | 13475-82-6 | Soil/Atmosphere | 0.02%/2.50% | Atmosphere-specific |
| 1,3-Bis(3-glycidoxypropyl)tetramethyldisiloxane | 126-80-7 | Soil/Atmosphere | 0.00%/2.12% | Atmosphere-specific |
| Pentadecanoic acid | 1002-84-2 | Soil/Atmosphere | 0.07%/2.11% | Atmosphere-specific |
| Ethyl(dimethyl)ethoxysilane | 18173-55-2 | Soil/Atmosphere | 0.00%/2.04% | Atmosphere-specific |
| Oleic acid | 112-80-1 | Soil/Atmosphere | 0.06%/1.54% | Atmosphere-specific |
| (3Z)-3-(hydroxymethylidene)-1,7,7-trimethylbicyclo[2.2.1]heptan-2-one | 15051-75-9 | Soil/Atmosphere | 0.00%/1.32% | Atmosphere-specific |
| 2,4-dimethyl-heptane | 2213-23-2 | Soil/Atmosphere | 0.06%/1.00% | Atmosphere-specific |

**Table S3.** Overview of RNA-seq read quality.

| **Samples** | **Raw Reads** | **Clean Reads** | **N Ratio(%)** | **Q30 Ratio(%)** | **GC Ratio(%)** | **Total Map** |
| --- | --- | --- | --- | --- | --- | --- |
| CK-1 | 51075724 | 46720646 | 0.01% | 95.50% | 54.95% | 95.22% |
| CK-2 | 54838126 | 50806536 | 0.01% | 95.97% | 54.94% | 94.88% |
| CK-3 | 52237464 | 44257558 | 0.01% | 95.95% | 54.49% | 95.31% |
| DMCH-1 | 51418822 | 43813552 | 0.01% | 95.24% | 54.16% | 93.59% |
| DMCH-2 | 53792580 | 46687044 | 0.01% | 95.79% | 54.19% | 93.74% |
| DMCH-3 | 55145174 | 50888478 | 0.01% | 96.25% | 55.27% | 95.25% |
| Nonanal-1 | 53239866 | 48456788 | 0.01% | 95.78% | 54.92% | 95.81% |
| Nonanal-2 | 46421672 | 42213684 | 0.01% | 95.56% | 54.76% | 95.41% |
| Nonanal-3 | 49975378 | 43946366 | 0.01% | 95.36% | 54.25% | 93.59% |

**Table S4.** Primers used for RT-qPCR.

| **Gene** | **Primers (5′-3′)** | **MW** | **GC Content (%)** | **TM (°C)** | **Transcript ID** |
| --- | --- | --- | --- | --- | --- |
| *CHS7*  *(VC83_05759)* | F: TCCAACACCATCTGCCGAAA  R: GCCTTAGTGCCGACTGAGAA | 6016  6143 | 50  55 | 57.8  59.9 | XM_024469373.1 |
| *CHS1*  *(VC83_06323)* | F: TAACCTGTTTCGTGCTCGCT  R: GAAGGTGGTGTTTAGGGCGA | 6051  6294 | 50  55 | 57.8  59.9 | XM_024469925.1 |
| *SP1*  *(VC83_00970)* | F: TAGCTCCTCCAACTACGGCT  R: GTCCACCAAGACCGATGAGG | 6014  6137 | 55  60 | 59.9  61.9 | XM_024464656.1 |
| *CDC42*  *(VC83_05979)* | F: AATGTGCGCGAAAAGTGGTT  R: CCAACTCCTTCGCCATCCTC | 6222  5910 | 45  60 | 55.8  61.9 | XM_024469586.1 |
| *STE12*  *(VC83_07778)* | F: GGAGGATTACGAAGGCGAGG  R: TTCGAGCCCCATCAAACTCC | 6297  5983 | 60  55 | 61.9  59.9 | XM_024471343.1 |
| *COX12*  *(VC83_03780)* | F: CGCTTCCCAAACCAGAACCA  R: CACTTGGGCACAATGATCGG | 6001  6143 | 55  55 | 59.9  59.9 | XM_024467423.1 |
| *QCR8*  *(VC83_03020)* | F: GGTCGGAAAGTACGGAAGGT  R: CAGGCCGTAGTAGACGATGC | 6272  6168 | 55  60 | 59.9  61.9 | XM_024466669.1 |
| *RPS7*  *(VC83_08116)* | F: CTCCGCCCTCTTCAATTCGT  R: GGAAACCCTGAAGGGATGGG | 5956  6257 | 55  60 | 59.9  61.9 | XM_024471677.1 |
| *RPS3*  *(VC83_05430)* | F: GGTTGCGAGGTTGTCGTTTC  R: TCGATGAAGTCCTTGGCTGG | 6187  6165 | 55  55 | 59.9  59.9 | XM_024469052.1 |
| *EFG1*  *(VC83_05289)* | F: GGACTACACCTTGAACCGCA  R: CATTGCCCATTGGTTCGTGG | 6072  6116 | 55  55 | 59.9  59.9 | XM_024468911.1 |

F, forward primer; R, reverse primer. *EFG1*, reference gene.

**Table S5.** Screening for DEGs encoding proteases with putative functions related to *P. destructans* virulence.

| **Gene^a^** | **Description** | **Blastx^b^** | **MeanTPM(T)^c^** | **MeanTPM(C)^d^** | **FDR^e^** | **Group^f^** |
| --- | --- | --- | --- | --- | --- | --- |
| **Subtilase-Family Proteases** | | |  |  |  |  |
| VC83_06062 | Subtilisin-like protease 2 | - | 4.37 | 19.01 | 0.00326136 | DMCH |
| VC83_06607 | Pheromone processing endoprotease | - | 12.25 | 39.70 | 0.000381991 | DMCH |
| VC83_04892 | Subtilisin-like protease 1 | - | 19.83 | 69.08 | 0.013004073 | DMCH |
| VC83_09074 | Subtilisin-like protease 3 | - | 5.81 | 0.19 | 4.94E-08 | Nonanal |
| VC83_02181 | Tripeptidyl-peptidase sed2 | SED2_ASPFU | 13.86 | 3.26 | 0.000431543 | Nonanal |
| **Aspartyl-Family Proteases** | |  |  |  |  |  |
| VC83_07794 | Probable aspartic-type endopeptidase opsB | OPSB_ASPOR | 1.57 | 9.25 | 0.000133045 | Nonanal |
| VC83_07794 | Probable aspartic-type endopeptidase opsB | OPSB_ASPOR | 2.65 | 9.25 | 0.002025992 | DMCH |
| VC83_06748 | Putative aspergillopepsin A-like aspartic endopeptidase AFUA_2G15950 | Y5950_ASPFU | 9.83 | 48.01 | 0.002713697 | Nonanal |
| VC83_06748 | Putative aspergillopepsin A-like aspartic endopeptidase AFUA_2G15950 | Y5950_ASPFU | 12.42 | 48.01 | 0.00179983 | DMCH |
| VC83_04754 | Vacuolar protease A | - | 372.81 | 1463.55 | 0.017594735 | Nonanal |
| VC83_07794 | Probable aspartic-type endopeptidase opsB | OPSB_ASPOR | 1.57 | 9.25 | 0.000133045 | Nonanal |
| VC83_06060 | Probable aspartic-type endopeptidase OPSB | OPSB_TRIVH | 32.10 | 150.66 | 0.028392448 | Nonanal |
| VC83_03986 | Probable aspartic-type endopeptidase CTSD | CTSD_TRIVH | 14.91 | 80.48 | 8.40E-05 | Nonanal |
| **Ubiquitin-specific-Family Protease** | |  |  |  |  |  |
| VC83_08320 | Probable ubiquitin carboxyl-terminal hydrolase 3 | UBP3_SCHPO | 23.43 | 104.26 | 0.023336236 | Nonanal |
| VC83_08320 | Probable ubiquitin carboxyl-terminal hydrolase 3 | UBP3_SCHPO | 28.60 | 104.26 | 0.017460155 | DMCH |
| VC83_05045 | Probable ubiquitin carboxyl-terminal hydrolase creB | CREB_ASPFU | 5.08 | 23.75 | 0.001647606 | Nonanal |
| VC83_05045 | Probable ubiquitin carboxyl-terminal hydrolase creB | CREB_ASPFU | 7.34 | 23.75 | 0.000934649 | DMCH |
| VC83_04639 | Ubiquitin carboxyl-terminal hydrolase 22-A | UB22A_XENLA | 10.80 | 33.70 | 0.011889736 | DMCH |
| **Other Protease** | |  |  |  |  |  |
| VC83_05359 | Cysteine protease | - | 7.41 | 21.29 | 0.021825546 | DMCH |
| VC83_05359 | Cysteine protease | - | 6.76 | 21.29 | 0.043855382 | Nonanal |
| VC83_03800 | Disintegrin and metalloproteinase domain-containing proteinB | ADMB_ASPFU | 27.05 | 146.44 | 0.000371261 | Nonanal |
| VC83_03800 | Disintegrin and metalloproteinase domain-containing proteinB | ADMB_ASPFU | 47.96 | 146.44 | 0.010099475 | DMCH |
| VC83_07049 | Bleomycin hydrolase | BLMH_HUMAN | 16.41 | 78.67 | 0.005174663 | Nonanal |
| VC83_05198 | Aspartyl aminopeptidase | DNPEP_ASPOR | 10.63 | 41.21 | 0.013096798 | Nonanal |
| VC83_05198 | Aspartyl aminopeptidase | DNPEP_ASPOR | 13.84 | 41.21 | 0.002956785 | DMCH |
| VC83_03174 | Calpain-8 | CAN8_MOUSE | 0.42 | 1.76 | 0.033566509 | Nonanal |
| VC83_02988 | CAAX prenyl protease 1 | STE24_YEAST | 95.33 | 278.81 | 0.005285994 | DMCH |

^a^ *P. destructans* genes (*Pseudogymnoascus destructans* strain:20631-21).

^b^ Hypothetical proteins were compared to the Swiss-Prot database using blastx, and only the results with the lowest E-value were chosen.

^c^ The mean Transcripts Per Million (TPM) of genes in the treatment groups.

^d^ The mean Transcripts Per Million (TPM) of genes in the control group.

^e^ Adjusted *p*-value of differential expression determined by DESeq2 after Benjamini-Hochberg FDR correction.

^f^ Treatment groups corresponding to the genes.

**Table S6.** Significantly enriched KEGG pathways of up- and downregulated DEGs for the DMCH and nonanal groups.

| **Pathway ID** | **KEGG-A-class** | **Pathway** | **Significant** | **Annotated** | ***p*-value** | **q-value** |
| --- | --- | --- | --- | --- | --- | --- |
| ko05171 | Human Diseases | Coronavirus disease - COVID-19 | 75/210 | 83/1939 | 9.86E-69 | 2.12E-66 |
| ko03010 | Genetic Information Processing | Ribosome | 78/210 | 108/1939 | 8.27E-57 | 8.90E-55 |
| ko04714 | Organismal Systems | Thermogenesis | 25/210 | 69/1939 | 1.07E-08 | 7.64E-07 |
| ko05415 | Human Diseases | Diabetic cardiomyopathy | 22/210 | 64/1939 | 2.61E-07 | 1.40E-05 |
| ko00190 | Metabolism | Oxidative phosphorylation | 24/210 | 76/1939 | 4.44E-07 | 1.91E-05 |
| ko05012 | Human Diseases | Parkinson disease | 29/210 | 105/1939 | 6.45E-07 | 2.31E-05 |
| ko05020 | Human Diseases | Prion disease | 28/210 | 105/1939 | 2.25E-06 | 6.91E-05 |
| ko05208 | Human Diseases | Chemical carcinogenesis - reactive oxygen species | 22/210 | 74/1939 | 4.37E-06 | 1.18E-04 |
| ko05010 | Human Diseases | Alzheimer disease | 28/210 | 110/1939 | 6.15E-06 | 1.47E-04 |
| ko04260 | Organismal Systems | Cardiac muscle contraction | 9/210 | 17/1939 | 1.95E-05 | 4.18E-04 |
| ko05016 | Human Diseases | Huntington disease | 30/210 | 130/1939 | 2.36E-05 | 4.60E-04 |
| ko05022 | Human Diseases | Pathways of neurodegeneration - multiple diseases | 29/210 | 130/1939 | 6.46E-05 | 1.16E-03 |
| ko05014 | Human Diseases | Amyotrophic lateral sclerosis | 27/210 | 132/1939 | 0.000546425 | 9.04E-03 |
| ko04932 | Human Diseases | Non-alcoholic fatty liver disease | 13/210 | 49/1939 | 0.001468131 | 2.25E-02 |
| ko04066 | Environmental Information Processing | HIF-1 signaling pathway | 6/210 | 18/1939 | 0.009109766 | 1.31E-01 |
| ko00920 | Metabolism | Sulfur metabolism | 4/210 | 13/1939 | 0.04356391 | 5.85E-01 |
| ko00521 | Metabolism | Streptomycin biosynthesis | 3/210 | 8/1939 | 0.04646606 | 5.88E-01 |

Significantly enriched KEGG pathways of upregulated DEGs of the DMCH treatment group.

| **Pathway ID** | **KEGG-A-class** | **Pathway** | **Significant** | **Annotated** | ***p*-value** | **q-value** |
| --- | --- | --- | --- | --- | --- | --- |
| ko04011 | Environmental Information Processing | MAPK signaling pathway - yeast | 26/229 | 57/1939 | 8.70E-11 | 2.48E-08 |
| ko03022 | Genetic Information Processing | Basal transcription factors | 13/229 | 31/1939 | 1.83E-05 | 2.61E-03 |
| ko04510 | Cellular Processes | Focal adhesion | 6/229 | 13/1939 | 0.002128834 | 1.92E-01 |
| ko04722 | Organismal Systems | Neurotrophin signaling pathway | 6/229 | 14/1939 | 0.003362226 | 1.92E-01 |
| ko05211 | Human Diseases | Renal cell carcinoma | 5/229 | 10/1939 | 0.003362666 | 1.92E-01 |
| ko04062 | Organismal Systems | Chemokine signaling pathway | 6/229 | 15/1939 | 0.005058576 | 2.06E-01 |
| ko04360 | Organismal Systems | Axon guidance | 6/229 | 15/1939 | 0.005058576 | 2.06E-01 |
| ko05203 | Human Diseases | Viral carcinogenesis | 10/229 | 36/1939 | 0.006787839 | 2.42E-01 |
| ko04660 | Organismal Systems | T cell receptor signaling pathway | 5/229 | 12/1939 | 0.008654968 | 2.74E-01 |
| ko04392 | Environmental Information Processing | Hippo signaling pathway - multiple species | 3/229 | 5/1939 | 0.01356442 | 3.51E-01 |
| ko04979 | Organismal Systems | Cholesterol metabolism | 3/229 | 5/1939 | 0.01356442 | 3.51E-01 |
| ko04664 | Organismal Systems | Fc epsilon RI signaling pathway | 4/229 | 9/1939 | 0.01475875 | 3.51E-01 |
| ko00520 | Metabolism | Amino sugar and nucleotide sugar metabolism | 10/229 | 41/1939 | 0.01758218 | 3.85E-01 |
| ko04213 | Organismal Systems | Longevity regulating pathway - multiple species | 8/229 | 31/1939 | 0.02347666 | 4.15E-01 |
| ko04010 | Environmental Information Processing | MAPK signaling pathway | 6/229 | 20/1939 | 0.02357502 | 4.15E-01 |
| ko03320 | Organismal Systems | PPAR signaling pathway | 5/229 | 15/1939 | 0.02438106 | 4.15E-01 |
| ko00592 | Metabolism | alpha-Linolenic acid metabolism | 3/229 | 6/1939 | 0.0247825 | 4.15E-01 |
| ko04936 | Human Diseases | Alcoholic liver disease | 5/229 | 16/1939 | 0.03214146 | 5.09E-01 |
| ko00591 | Metabolism | Linoleic acid metabolism | 2/229 | 3/1939 | 0.0384265 | 5.38E-01 |
| ko02025 | Cellular Processes | Biofilm formation - Pseudomonas aeruginosa | 2/229 | 3/1939 | 0.0384265 | 5.38E-01 |
| ko04622 | Organismal Systems | RIG-I-like receptor signaling pathway | 3/229 | 7/1939 | 0.03964224 | 5.38E-01 |
| ko04014 | Environmental Information Processing | Ras signaling pathway | 6/229 | 23/1939 | 0.04542318 | 5.53E-01 |

Significantly enriched KEGG pathways of upregulated DEGs of the nonanal treatment group.

| **Pathway ID** | **KEGG-A-class** | **Pathway** | **Significant** | **Annotated** | ***p*-value** | **q-value** |
| --- | --- | --- | --- | --- | --- | --- |
| ko05171 | Human Diseases | Coronavirus disease - COVID-19 | 76/146 | 83/1939 | 1.93E-86 | 3.07E-84 |
| ko03010 | Genetic Information Processing | Ribosome | 79/146 | 108/1939 | 1.79E-74 | 1.42E-72 |
| ko04714 | Organismal Systems | Thermogenesis | 13/146 | 69/1939 | 0.001410397 | 7.48E-02 |
| ko04260 | Organismal Systems | Cardiac muscle contraction | 5/146 | 17/1939 | 0.00666418 | 2.65E-01 |
| ko04932 | Human Diseases | Non-alcoholic fatty liver disease | 9/146 | 49/1939 | 0.009195583 | 2.67E-01 |
| ko05012 | Human Diseases | Parkinson disease | 15/146 | 105/1939 | 0.01008476 | 2.67E-01 |
| ko05020 | Human Diseases | Prion disease | 14/146 | 105/1939 | 0.02269546 | 4.80E-01 |
| ko00190 | Metabolism | Oxidative phosphorylation | 11/146 | 76/1939 | 0.024156 | 4.80E-01 |
| ko05010 | Human Diseases | Alzheimer disease | 14/146 | 110/1939 | 0.0326598 | 5.77E-01 |
| ko05415 | Human Diseases | Diabetic cardiomyopathy | 9/146 | 64/1939 | 0.04682162 | 6.58E-01 |
| ko05208 | Human Diseases | Chemical carcinogenesis - reactive oxygen species | 10/146 | 74/1939 | 0.04700802 | 6.58E-01 |

Significantly enriched KEGG pathways of downregulated DEGs of the DMCH treatment group.

| **Pathway ID** | **KEGG-A-class** | **Pathway** | **Significant** | **Annotated** | ***p*-value** | **q-value** |
| --- | --- | --- | --- | --- | --- | --- |
| ko04011 | Environmental Information Processing | MAPK signaling pathway - yeast | 21/146 | 57/1939 | 1.32E-10 | 2.81E-08 |
| ko04510 | Cellular Processes | Focal adhesion | 5/146 | 13/1939 | 0.001774285 | 1.39E-01 |
| ko04070 | Environmental Information Processing | Phosphatidylinositol signaling system | 6/146 | 19/1939 | 0.001962079 | 1.39E-01 |
| ko04933 | Human Diseases | AGE-RAGE signaling pathway in diabetic complications | 4/146 | 9/1939 | 0.002884329 | 1.53E-01 |
| ko04138 | Cellular Processes | Autophagy - yeast | 9/146 | 44/1939 | 0.004391911 | 1.86E-01 |
| ko00592 | Metabolism | alpha-Linolenic acid metabolism | 3/146 | 6/1939 | 0.007064286 | 2.27E-01 |
| ko04120 | Genetic Information Processing | Ubiquitin mediated proteolysis | 10/146 | 56/1939 | 0.007510462 | 2.27E-01 |
| ko00562 | Metabolism | Inositol phosphate metabolism | 5/146 | 19/1939 | 0.01107555 | 2.58E-01 |
| ko05131 | Human Diseases | Shigellosis | 8/146 | 42/1939 | 0.01114557 | 2.58E-01 |
| ko05417 | Human Diseases | Lipid and atherosclerosis | 6/146 | 27/1939 | 0.01294291 | 2.58E-01 |
| ko02025 | Cellular Processes | Biofilm formation - Pseudomonas aeruginosa | 2/146 | 3/1939 | 0.0160633 | 2.58E-01 |
| ko04722 | Organismal Systems | Neurotrophin signaling pathway | 4/146 | 14/1939 | 0.01701807 | 2.58E-01 |
| ko00561 | Metabolism | Glycerolipid metabolism | 5/146 | 21/1939 | 0.01715019 | 2.58E-01 |
| ko04139 | Cellular Processes | Mitophagy - yeast | 7/146 | 38/1939 | 0.02064253 | 2.58E-01 |
| ko00563 | Metabolism | Glycosylphosphatidylinositol (GPI)-anchor biosynthesis | 5/146 | 22/1939 | 0.02088108 | 2.58E-01 |
| ko03320 | Organismal Systems | PPAR signaling pathway | 4/146 | 15/1939 | 0.02187616 | 2.58E-01 |
| ko04360 | Organismal Systems | Axon guidance | 4/146 | 15/1939 | 0.02187616 | 2.58E-01 |
| ko05135 | Human Diseases | Yersinia infection | 4/146 | 15/1939 | 0.02187616 | 2.58E-01 |
| ko04113 | Cellular Processes | Meiosis - yeast | 9/146 | 59/1939 | 0.02945949 | 3.29E-01 |
| ko05211 | Human Diseases | Renal cell carcinoma | 3/146 | 10/1939 | 0.03387434 | 3.59E-01 |
| ko04392 | Environmental Information Processing | Hippo signaling pathway - multiple species | 2/146 | 5/1939 | 0.04841507 | 4.69E-01 |
| ko05203 | Human Diseases | Viral carcinogenesis | 6/146 | 36/1939 | 0.04869583 | 4.69E-01 |

Significantly enriched KEGG pathways of downregulated DEGs of the nonanal treatment group.

**Table S7.** Significantly enriched KEGG pathways for DEMs in the DMCH and nonanal groups.

| **Pathway ID** | **Pathway_Hierarchy1** | **Pathway_Hierarchy2** | **Pathway** | ***p*-value** | **q-value** | **Rich Factor** |
| --- | --- | --- | --- | --- | --- | --- |
| ko02010 | Environmental Information Processing | Membrane transport | ABC transporters | 7.69316E-11 | 1.43093E-08 | 0.218978102 |
| ko01230 | Metabolism | Global and overview maps | Biosynthesis of amino acids | 1.57201E-07 | 1.09743E-05 | 0.1875 |
| ko04974 | Organismal Systems | Digestive system | Protein digestion and absorption | 1.77004E-07 | 1.09743E-05 | 0.29787234 |
| ko00970 | Genetic Information Processing | Translation | Aminoacyl-tRNA biosynthesis | 0.000127099 | 0.005910094 | 0.211538462 |
| ko02060 | Environmental Information Processing | Membrane transport | Phosphotransferase system (PTS) | 0.000300458 | 0.009546022 | 0.192982456 |
| ko04742 | Organismal Systems | Sensory system | Taste transduction | 0.000320532 | 0.009546022 | 0.25 |
| ko04024 | Environmental Information Processing | Signal transduction | cAMP signaling pathway | 0.000359259 | 0.009546022 | 0.28 |
| ko04080 | Environmental Information Processing | Signaling molecules and interaction | Neuroactive ligand-receptor interaction | 0.000580364 | 0.012903462 | 0.192307692 |
| ko04150 | Environmental Information Processing | Signal transduction | mTOR signaling pathway | 0.000701554 | 0.012903462 | 0.75 |
| ko04923 | Organismal Systems | Endocrine system | Regulation of lipolysis in adipocytes | 0.000759169 | 0.012903462 | 0.357142857 |
| ko00250 | Metabolism | Amino acid metabolism | Alanine, aspartate and glutamate metabolism | 0.000763108 | 0.012903462 | 0.25 |
| ko00290 | Metabolism | Amino acid metabolism | Valine, leucine and isoleucine biosynthesis | 0.001440046 | 0.020749495 | 0.260869565 |
| ko01210 | Metabolism | Global and overview maps | 2-Oxocarboxylic acid metabolism | 0.001450234 | 0.020749495 | 0.126865672 |
| ko04917 | Organismal Systems | Endocrine system | Prolactin signaling pathway | 0.002476255 | 0.032898811 | 0.363636364 |
| ko04978 | Organismal Systems | Digestive system | Mineral absorption | 0.005074799 | 0.062927505 | 0.206896552 |
| ko00261 | Metabolism | Biosynthesis of other secondary metabolites | Monobactam biosynthesis | 0.005779128 | 0.06718236 | 0.179487179 |
| ko00220 | Metabolism | Amino acid metabolism | Arginine biosynthesis | 0.008354358 | 0.087720584 | 0.217391304 |
| ko00997 | Metabolism | Biosynthesis of other secondary metabolites | Biosynthesis of various secondary metabolites - part 3 | 0.008489089 | 0.087720584 | 0.153846154 |
| ko04727 | Organismal Systems | Nervous system | GABAergic synapse | 0.011888643 | 0.116383562 | 0.333333333 |
| ko04973 | Organismal Systems | Digestive system | Carbohydrate digestion and absorption | 0.016636289 | 0.147349986 | 0.185185185 |
| ko00073 | Metabolism | Lipid metabolism | Cutin, suberine and wax biosynthesis | 0.016636289 | 0.147349986 | 0.185185185 |
| ko04723 | Organismal Systems | Nervous system | Retrograde endocannabinoid signaling | 0.020269508 | 0.17136948 | 0.210526316 |
| ko00260 | Metabolism | Amino acid metabolism | Glycine, serine and threonine metabolism | 0.022007981 | 0.172869034 | 0.14 |
| ko04216 | Cellular Processes | Cell growth and death | Ferroptosis | 0.022305682 | 0.172869034 | 0.172413793 |
| ko00140 | Metabolism | Lipid metabolism | Steroid hormone biosynthesis | 0.024386794 | 0.175390797 | 0.111111111 |
| ko00270 | Metabolism | Amino acid metabolism | Cysteine and methionine metabolism | 0.02546878 | 0.175390797 | 0.126984127 |
| ko04927 | Organismal Systems | Endocrine system | Cortisol synthesis and secretion | 0.027419862 | 0.175390797 | 0.25 |
| ko04721 | Organismal Systems | Nervous system | Synaptic vesicle cycle | 0.027419862 | 0.175390797 | 0.25 |
| ko04068 | Environmental Information Processing | Signal transduction | FoxO signaling pathway | 0.028830917 | 0.175390797 | 0.4 |
| ko00620 | Metabolism | Carbohydrate metabolism | Pyruvate metabolism | 0.029098163 | 0.175390797 | 0.161290323 |
| ko02020 | Environmental Information Processing | Signal transduction | Two-component system | 0.029394855 | 0.175390797 | 0.132075472 |
| ko00650 | Metabolism | Carbohydrate metabolism | Butanoate metabolism | 0.030174761 | 0.175390797 | 0.142857143 |
| ko00350 | Metabolism | Amino acid metabolism | Tyrosine metabolism | 0.032129708 | 0.181094721 | 0.115384615 |
| ko04925 | Organismal Systems | Endocrine system | Aldosterone synthesis and secretion | 0.033463559 | 0.18306535 | 0.181818182 |
| ko00460 | Metabolism | Metabolism of other amino acids | Cyanoamino acid metabolism | 0.040677449 | 0.216171587 | 0.133333333 |
| ko04913 | Organismal Systems | Endocrine system | Ovarian steroidogenesis | 0.044485361 | 0.22984103 | 0.166666667 |
| ko00300 | Metabolism | Amino acid metabolism | Lysine biosynthesis | 0.046261875 | 0.232559697 | 0.142857143 |
| ko02010 | Environmental Information Processing | Membrane transport | ABC transporters | 7.69316E-11 | 1.43093E-08 | 0.218978102 |

Significantly enriched KEGG pathways of DEMs in the DMCH treatment group.

| **Pathway ID** | **Pathway_Hierarchy1** | **Pathway_Hierarchy2** | **Pathway** | ***p*-value** | **q-value** | **Rich Factor** |
| --- | --- | --- | --- | --- | --- | --- |
| ko02010 | Environmental Information Processing | Membrane transport | ABC transporters | 4.12493E-10 | 6.97113E-08 | 0.204379562 |
| ko04974 | Organismal Systems | Digestive system | Protein digestion and absorption | 4.19457E-06 | 0.000354441 | 0.255319149 |
| ko01230 | Metabolism | Global and overview maps | Biosynthesis of amino acids | 4.11895E-05 | 0.002320341 | 0.1484375 |
| ko04150 | Environmental Information Processing | Signal transduction | mTOR signaling pathway | 0.000579328 | 0.024476623 | 0.75 |
| ko02060 | Environmental Information Processing | Membrane transport | Phosphotransferase system (PTS) | 0.000747214 | 0.025255834 | 0.175438596 |
| ko00970 | Genetic Information Processing | Translation | Aminoacyl-tRNA biosynthesis | 0.001510261 | 0.039789627 | 0.173076923 |
| ko04024 | Environmental Information Processing | Signal transduction | cAMP signaling pathway | 0.001648091 | 0.039789627 | 0.24 |
| ko04978 | Organismal Systems | Digestive system | Mineral absorption | 0.003691113 | 0.077974757 | 0.206896552 |
| ko04923 | Organismal Systems | Endocrine system | Regulation of lipolysis in adipocytes | 0.005207944 | 0.097793611 | 0.285714286 |
| ko04742 | Organismal Systems | Sensory system | Taste transduction | 0.006153936 | 0.104001526 | 0.1875 |
| ko04973 | Organismal Systems | Digestive system | Carbohydrate digestion and absorption | 0.012847615 | 0.197386083 | 0.185185185 |
| ko00140 | Metabolism | Lipid metabolism | Steroid hormone biosynthesis | 0.01585735 | 0.21071798 | 0.111111111 |
| ko00261 | Metabolism | Biosynthesis of other secondary metabolites | Monobactam biosynthesis | 0.016209075 | 0.21071798 | 0.153846154 |
| ko00270 | Metabolism | Amino acid metabolism | Cysteine and methionine metabolism | 0.018012334 | 0.217434603 | 0.126984127 |
| ko04080 | Environmental Information Processing | Signaling molecules and interaction | Neuroactive ligand-receptor interaction | 0.019541348 | 0.220165851 | 0.134615385 |
| ko00620 | Metabolism | Carbohydrate metabolism | Pyruvate metabolism | 0.02273149 | 0.230028384 | 0.161290323 |
| ko04927 | Organismal Systems | Endocrine system | Cortisol synthesis and secretion | 0.02313895 | 0.230028384 | 0.25 |
| ko04925 | Organismal Systems | Endocrine system | Aldosterone synthesis and secretion | 0.027189717 | 0.255281236 | 0.181818182 |
| ko00290 | Metabolism | Amino acid metabolism | Valine, leucine and isoleucine biosynthesis | 0.031571381 | 0.266778167 | 0.173913043 |
| ko00220 | Metabolism | Amino acid metabolism | Arginine biosynthesis | 0.031571381 | 0.266778167 | 0.173913043 |
| ko04913 | Organismal Systems | Endocrine system | Ovarian steroidogenesis | 0.036343643 | 0.280777697 | 0.166666667 |
| ko00300 | Metabolism | Amino acid metabolism | Lysine biosynthesis | 0.036550943 | 0.280777697 | 0.142857143 |
| ko00360 | Metabolism | Amino acid metabolism | Phenylalanine metabolism | 0.039369412 | 0.289279594 | 0.116666667 |
| ko00260 | Metabolism | Amino acid metabolism | Glycine, serine and threonine metabolism | 0.048575763 | 0.342054333 | 0.12 |

Significantly enriched KEGG pathways of DEMs in the nonanal treatment group.
